# Supplementary material for: Cancer gene mutation frequencies for the U.S. population
Source: Nat Commun. 2021 Oct 13;12:5961. doi: 10.1038/s41467-021-26213-y (PMC8514428; doi:10.1038/s41467-021-26213-y)
Supplement: Supplementary file 1 — Supplementary Information [file 41467_2021_26213_MOESM1_ESM.pdf]

Supplementary Information

Cancer gene mutation frequencies for the U.S. population

Gaurav Mendiratta, Eugene Ke, Meraj Aziz,
David Liarakos, Melinda Tong, Edward C. Stites

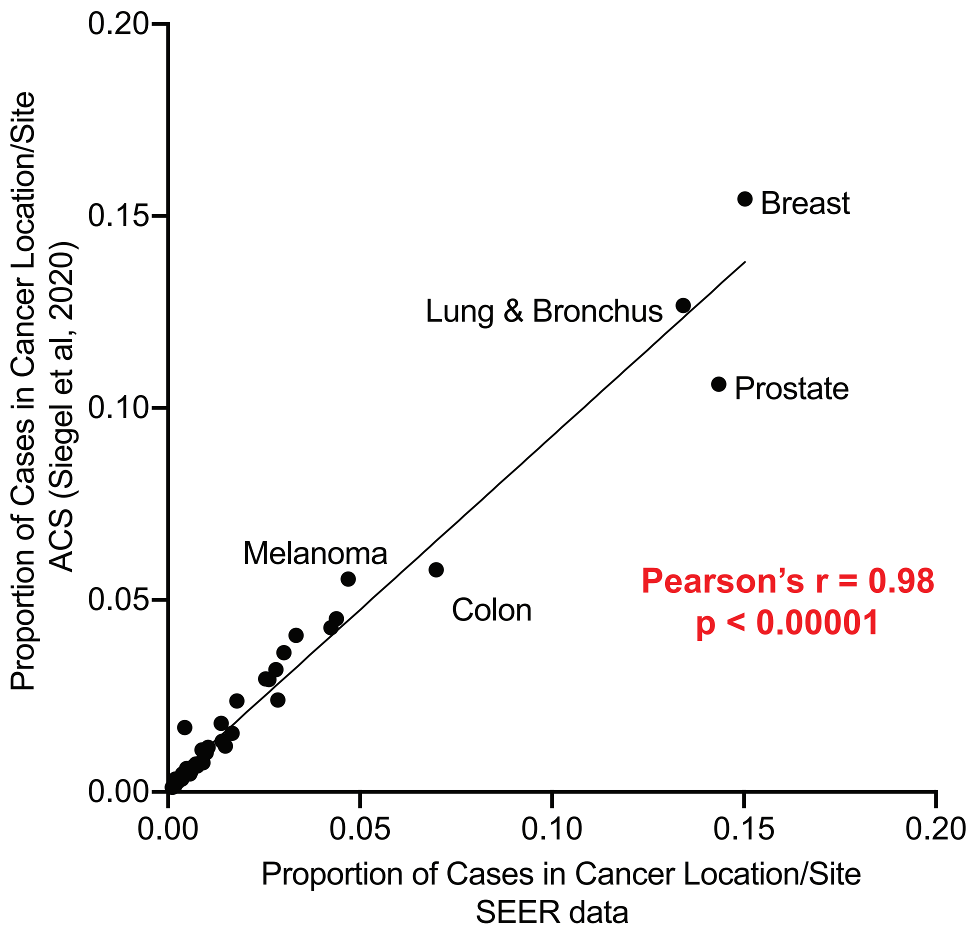

Figure S1. Comparison of SEER and ACS cancer statistics. Rates of cancer by location were compared between the annual ACS cancer epidemiology study [35] and the ROSETTA-processed SEER dataset that we utilized. The comparison found a strong agreement between the data, with a Pearson's r value of 0.98 and p-value (2-tailed) ($p < 0.00001$) (line of regression shown in gray).

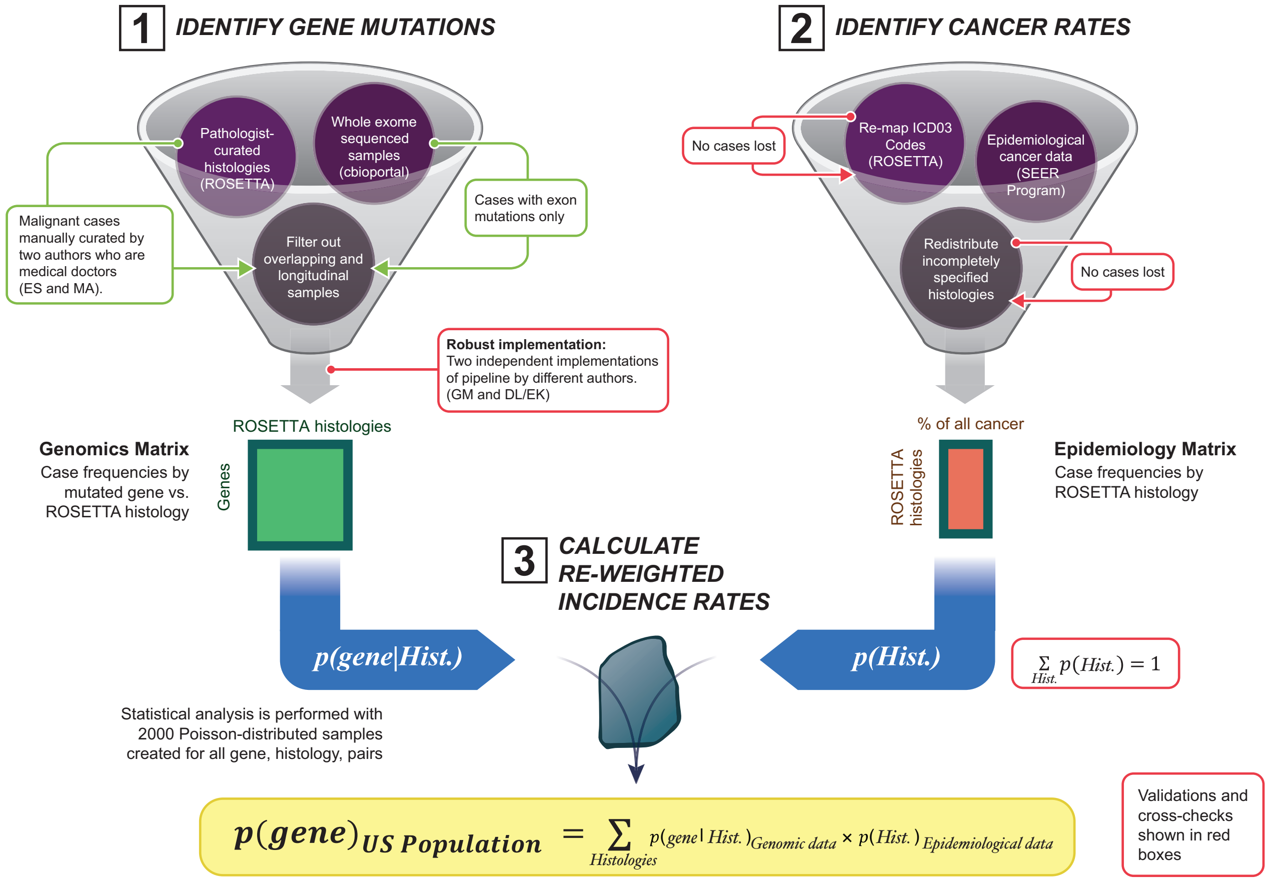

Figure S2. Schematic of the data analysis process. Step one involves processing cancer genomic data on the basis of available metadata into ROSETTA histological categories. This results in a matrix where each row lists the count of sequenced samples with a mutation in that gene for each ROSETTA category with representative sequencing data (with each column representing a different ROSETTA category). Step two involves obtaining a matrix that lists the proportion of all cancers that is due to each ROSETTA category (the rows). Multiple quality control checks are employed throughout step one and step two (indicated in red boxes). The resulting matrices specify the probabilities of a gene having a mutation within each ROSETTA histology, and the probability that a cancer is associated with the specified ROSETTA histology. The product of these two matrices results in a column vector where each row is one of the different genes, and the value in the vector for each gene is the estimated proportion of all new cancer cases with a mutation in that gene.

Supplementary Methods: Explanation of ROSETTA mapping and utilization

ROSETTA as applied to genomic data

We created ROSETTA to enable the mapping of similar cancer types between cancer genomic and cancer epidemiologic data. The ROSETTA categories were determined with consideration of the different nomenclatures used in epidemiology and cancer genomics. ROSETTA category definitions were also influenced by the types of information included in sample metadata from cancer genomic studies.

For each patient sample in a considered cancer genomics study, we utilized the metadata associated with each sample to assign it to a ROSETTA category. Genomic metadata is commonly organized as a table, where the different rows correspond to the different samples, and the different columns include different information about the sample. If sample metadata provided histological classification, such as an ICD-O-3 morphology, this could enable a finer subdivision of cancer types within a single study. For example, a genomics study of non-small cell lung cancer could be divided into lung adenocarcinoma and lung squamous cell carcinoma on the basis of the histological metadata provided for each sample. If a study focused on the same general cancer histology in nearby and distinguishable locations, subdivision based on the anatomical location was possible. For example, if a study included both esophageal and gastric adenocarcinoma and the sample data included the location of the tumor, these samples could be annotated as esophageal adenocarcinoma or as gastric adenocarcinoma. When metadata within a study was incomplete, we would only include the samples with the required metadata for consistent classification across all samples. If a study was focused upon one type of cancer and either could not be subdivided, and/or we decided that subdivision was not indicated to keep our practices consistent across the different genomic studies for that type of cancer, we would assign all cancers within a study to the same ROSETTA code. Figure S3 below highlights these three different situations.

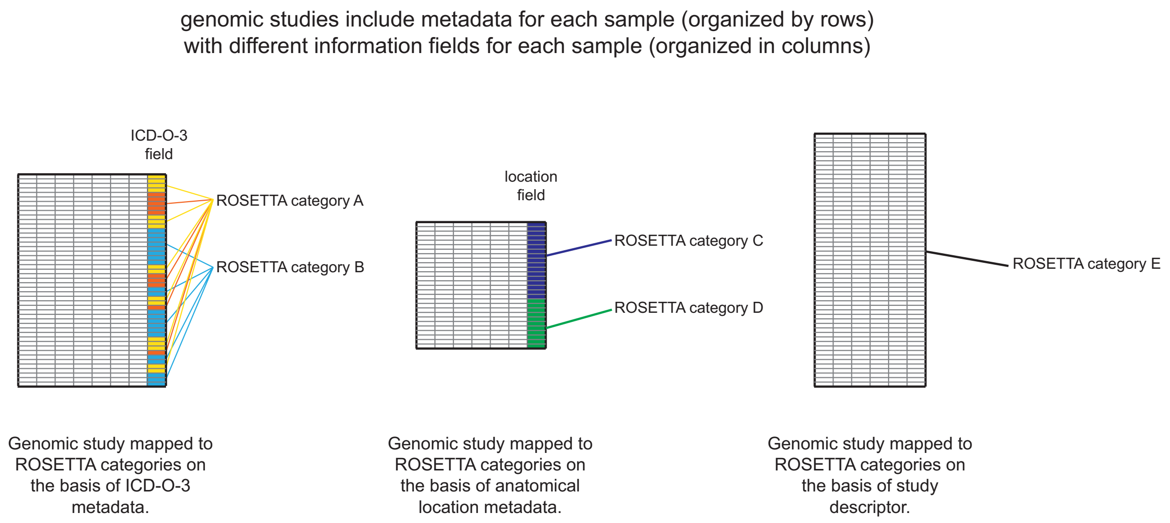

Figure S3. Mapping of genomic studies to ROSETTA Categories

ROSETTA as applied to SEER epidemiological data

Outputs from SEER are commonly formatted as a table, where the rows indicate anatomical locations and the columns indicate histological codes. Both ICD-O-3 location and morphology (histology) codes are grouped for similar anatomical structures and for similar major histologies, respectively. Many cancer genomic studies appear to have sample inclusion decisions that spanned a range of similar anatomical and/or histological groupings. For example, head and neck squamous cell carcinoma (SCC) would appear to span the ICD-O-3 anatomical location codes that describe specific areas of the head and neck, and also the ICD-O-3 histological codes that describe the various types of SCC. Similarly, lung SCC would appear to span the same ICD-O-3 histological codes for SCC, but the ICD-O-3 anatomical location codes would describe the different areas of the lung. In cases like this, we would specify a range of ICD-O-3 anatomical and histological codes that corresponded to a single ROSETTA code. Figure S4 shows examples for head and neck SCC and for lung SCC.

Some ICD-O-3 histological codes are used only for one form of cancer. For example, there are highly specific, individual, ICD-O-3 codes for cholangiocarcinoma (81603), renal cell carcinoma (83173), and acral lentiginous melanoma (87443). When codes exist that were clearly indicated for, and utilized for, only one type of cancer, we mapped that entire column to a ROSETTA code specific for that form of cancer. Figure S4 shows an example below for cholangiocarcinoma and for acral (lentiginous) melanoma.

Several forms of adenocarcinoma were defined in a regional manner, similar to what was described for the SCC examples detailed earlier in this document. For example, lung adenocarcinoma and colorectal adenocarcinoma were mapped in this approach of regions of neighboring ICD-O-3 histology and location codes. However, if one of the highly specific, individual, ICD-O-3 codes fell within this grouping of ICD-O-3 histology codes, those cases were only counted for their more specific classification. Figure S4 shows an example for lung adenocarcinoma and colorectal adenocarcinoma, where the color scheme highlights that if any cancer had been characterized in the SEER dataset as cholangiocarcinoma with a lung or colorectal anatomical location, it would still be counted as cholangiocarcinoma. (Such atypically pairs of histology and location code were uncommon, so the handling of such situations is unlikely to have a meaningful impact on calculated mutation proportions.)

Some forms of adenocarcinoma might be further subdivided. For example, pancreatic neuroendocrine cancers have distinct genomic characteristics from pancreatic adenocarcinoma, but the neuroendocrine cancer ICD-O-3 codes are included within the codes for adenocarcinoma. Acinar cell carcinoma of the pancreas is another example of a distinguishable subset of pancreatic adenocarcinoma known to have distinct genomics. In situations like this, we would further subdivide the regional adenocarcinoma. For example, Figure S4 shows an example where we subdivide the subsection of the matrix that includes data for the pancreas and for adenocarcinomas into distinct subsets for pancreatic neuroendocrine tumor, acinar cell carcinoma of the pancreas, and pancreatic adenocarcinoma.

Anatomical locations can also indicate a unique subset of cancer. For example, uveal melanoma has very distinct genetics from other forms of melanoma. However, there is no unique ICD-O-3 histology code for uveal melanoma. Thus, we defined it as a melanoma that with a site of origin ICD-O-3 topography code that specified the eye or orbit. Figure S4 highlights how melanoma histology codes were subdivided into acral melanoma (by histology code), into uveal melanoma (by anatomical location code), and malignant melanoma (for all remaining cases with a malignant melanoma histology code).

Within any subsection of the SEER output matrix that maps to the same ROSETTA code, the total number of cases from the corresponding ICD-O-3 code pairs would be assigned to the new ROSETTA code.

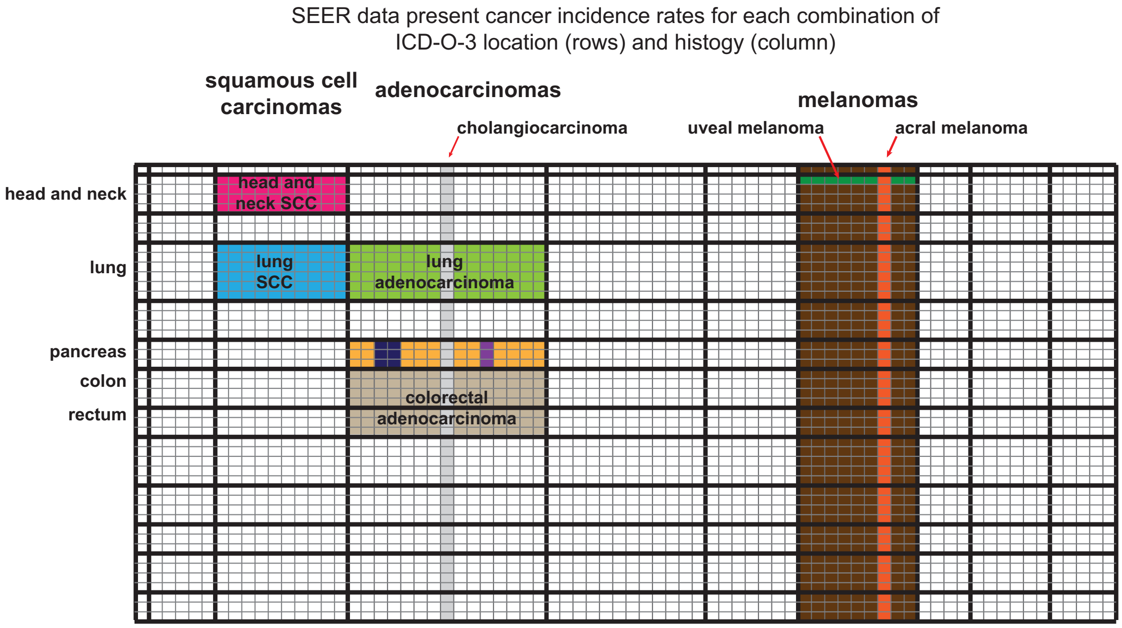

Figure S4. Mapping of SEER epidemiological data to ROSETTA Categories

Implementation of ROSETTA to cancer genomics

Patient sample information, including metadata, was downloaded for the different cancer exome sequencing studies considered. Each of the samples was then assigned to a single ROSETTA code, provided that it had not been propagated as a cell line or xenograft before sequencing and provided that the necessary location and/or histological metadata was available. Samples were assigned by manual curation of the metadata. Supplementary Table 2 lists all exome sequencing studies considered. Supplementary Table 2 also lists all ROSETTA codes that were assigned to each study.

The text files for each genomic study are included in the supplementary code. These text documents provide the mapping from each sample to a single ROSETTA code. These files were utilized during genomic data analysis to attribute the mutations from each sample to the correct ROSETTA code.

The output of the processing of all of the genomic data files is a matrix, where each row is a different gene, each column is a different ROSETTA category, and the values in the matrix are the number of samples that had the corresponding gene mutated within the corresponding ROSETTA category. A total number of samples sequenced for each ROSETTA code was also recorded to allow conversion of counts to proportions. Additional details on the matrix and calculation are provided in a later section of this document.

Implementation of ROSETTA to epidemiological data

The SEER output matrix was first processed to account for tumors of unclear anatomical location. Whenever a sample in the SEER data included a specific histology code, but utilized the ICD-O-3 location code to indicate the location was unknown, we assume that the tumor came from one of the other, specific, locations with a probability proportional to the count of cases from the other locations for this specific histology. For each ICD-O-3 histology, we distributed the total number of tumors recorded to have come from an unknown location to the other locations in a manner proportional to the count of cases from the other locations.

The SEER output matrix was next processed to account for tumors of unclear histological type. Whenever the ICD-O-3 histology code specified a generic malignant histology (i.e. Neoplasms NOS; codes <80053), we assume that the tumor had a specific cancer histology and that we could estimate the actual histology on the basis of the characterized tumors from the same location. Within each specific location, we distributed the generic malignant neoplasm counts to the other ICD-O-3 histologies within the same location with probabilities proportional to the count of cases from the other histologies.

We then implemented our ROSETTA reclassification of the specific ICD-O-3 location and histology codes to ROSETTA codes. A document that lists all regroupings is provided with the supplementary software, along with a script for processing ROSETTA reclassification of the SEER output matrix. (Note: ROSETTA utilizes some ICD-O-3 morphology codes as-is when reassignment is not necessary, either due to the high specificity of the ICD-O-3 code for a single form of cancer, or due to an absence of exome sequencing data for that cancer classification.) There is very good correlation between the counts of tumors at different locations in our post-ROSETTA processed data and the counts from the annual American Cancer Society's cancer epidemiology report (Figure S1). This provides an additional comparison that helps support the validity of our processing.

Sample calculation of epidemiologically weighted mutation proportions

The first step in our process of obtaining epidemiologically weighted mutation proportions for cancer associated gene mutations is the analysis of exome sequencing data. Our processing of genomic data results in a m x n matrix, where m is number of genes reported to be mutated in at least one sample (m = 21,271) and n is number of ROSETTA categories for which genomic sequencing data was available (n = 73). We use C to refer to this matrix of cancer genomic data. Within Figure S5 we demonstrate the matrix formalism with a simplified 3 x 3 matrix where we focus on only three genes and three forms of cancer. (In this toy example, we denote the three types of cancer and three genes nonspecifically to prevent comparisons with specific cancers and specific genes.) Each row specifies a different gene, and each column specifies a different form of cancer for which sequencing data is available. At the intersection of gene and cancer type indices is the proportion of all sequenced patient samples for that type of cancer that harbor one or more mutations to the indicated gene. Individual values in the matrix can therefore not exceed 100% or be less than 0%. The sum of a column and the sum of a row have no additional constraints. Within our main study, the values that populate the matrix are identified on the basis of existing cancer genomic studies.

Figure S5. Simplified example of a genomic matrix, *C*, with three genes and three types of cancer.

C

	cancer 1	cancer 2	cancer 3
gene 1	38%	21%	53%
gene 2	17%	38%	7%
gene 3	2%	7%	12%

The second step in our process of obtaining epidemiologically weighted mutation proportions for cancer associated gene mutations is the analysis of epidemiological data. Our processing of SEER epidemiological data results in a *k* x *1* matrix that includes all types of cancers, and where *k* is the total number of ROSETTA categories needed to describe all cancer cases in the epidemiological database (*k* = 370). We use *S* to refer to this matrix of processed SEER data. The values in *k* are the proportion of all cancers that are that form of cancer. The sum of this vector necessarily equals 100%.

Sequencing data is not available for all forms of cancer. Therefore, the value of *k* (the number of ROSETTA codes needed to describe cases in SEER epidemiological data) is greater than *n* (the number of ROSETTA codes needed to describe available exome sequencing data). Of note, 93% of total cases within SEER data are included within the 73 ROSETTA codes represented in genomic data. We therefore limit our integration of epidemiological data and genomic data to the 93% of cancer cases that have representative genomic data. To do this, we create the *S'* matrix, which is an *n* x *1* matrix that is limited to the forms of cancer for which sequencing data is present. (Reminder: *n* is number of ROSETTA categories for which genomic sequencing data was available, and *n* = 73). We rescale all proportions so that they sum to 100%; once our calculation is final this rescaling effectively implements the assumption that the weighted average of sequenced cancers is a good estimate of both the sequenced and unsequenced cancers. Figure S6 shows a simplified example of an *S* and an *S'* matrix.

Figure S6. Simplified example of an epidemiology matrix, *S*, with four forms of cancer (indicated as four different colors). The *S* matrix is converted to the *S'* matrix, which reprents cancer types that have been seqeunced.

S

	proportion
cancer 1	52%
cancer 2	24%
cancer 3	17%
cancer 4	7%

S'

	proportion
cancer 1	55.9%
cancer 2	25.8%
cancer 3	18.3%

The third step in our process for obtaining epidemiologically weighted mutation proportions for cancer associated gene mutations is the multiplication of the *C* and *S'* matrices. The product of the *C* and *S'* matrices, or *C* x *S'*, is an *m* x *1* matrix. (Reminder: *m* is number of genes reported to have a mutation from the aggregated sequencing data, and *m* = 21,271). We refer to this matrix as *G*, as it provides the epidemiologically weighted gene mutation proportions. As in *C*, the *m* rows each correspond to a single gene. Like *C*, and as opposed to *S* and *S'*, the sums of the columns of *G* do not have to equal 100% because any gene is free to be mutated (or not) independently from the mutation status of the each of other genes. Figure S7 shows a sample matrix calculation based on the simplified sample *C* and sample *S'* matrices.

Figure S7. Simplified example of the calculated matrix of epidemiologically weighted gene mutation proportions, that is obtained by multiplying matrix *C* by matrix *S'*.

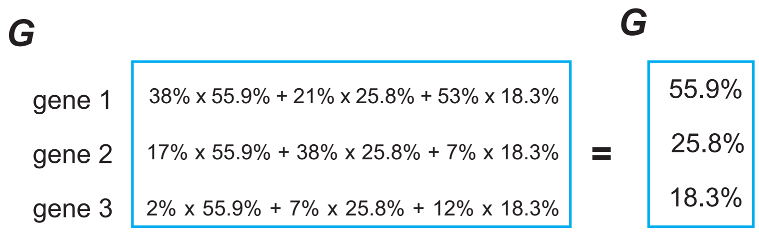

Implementation Notes

Our manual annotations of each cancer sample to a unique ROSETTA code is provided in the Supplementary Software. Within the folder \Genomics_Analysis\clinical there are sub-folders for each of the cancer descriptors that have been utilized for the parent data. Within each is a sub-sub-folder for each utilized genomics study within that cancer descriptor. Each sub-sub folder contains a text file that provides the ROSETTA annotation for each sequenced sample within that study. ROSETTA annotations for genomic studies were manually performed by co-authors ES and MA. Manual annotation would need to be performed for new studies, taking into strong consideration the annotation of related genomics studies to ensure consistency across samples.

Code that processes the ROSETTA annotations and combines them with mutation data from genomics studies is provided in the Supplementary Software 1:
\Genomics_Analysis\Genomics_Clinical_Mutations_Counter.ipynb

Code for our process of grouping SEER data from ICD-O-3 histology and location codes into ROSETTA categories is provided in the Supplementary Software:
\SEER_Analysis\SEER_python_code_RUNME.py

The file README.txt in the supplementary software provides instructions for running the source code, which was written in python 3.8.5 using the Jupyter notebook format. Required libraries are listed in the README.txt file, along with instructions for obtaining them.
