# Supplementary material for: Cancer gene mutation frequencies for the U.S. population
Source: Nat Commun. 2021 Oct 13;12:5961. doi: 10.1038/s41467-021-26213-y (PMC8514428; doi:10.1038/s41467-021-26213-y)
Supplement: Supplementary file 3 — Description of Additional Supplementary Files [file 41467_2021_26213_MOESM3_ESM.pdf]

Description of Additional Supplementary Files

File Name: Supplementary Data 1
Description: ROSETTA cancer types and relative abundances

File Name: Supplementary Data 2
Description: Genomic studies incorporated into this study

File Name: Supplementary Data 3
Description: Epidemiologically weighted mutation proportions across all cancers

File Name: Supplementary Data 4
Description: Epidemiologically weighted mutation proportions for adenocarcinomas, squamous cell carcinomas, melanomas, and transitional cell carcinoma

File Name: Supplementary Software 1
Description: Zipped source code for the analyses presented.
