# Supplementary material for: Cancer gene mutation frequencies for the U.S. population
Source: Nat Commun. 2021 Oct 13;12:5961. doi: 10.1038/s41467-021-26213-y (PMC8514428; doi:10.1038/s41467-021-26213-y)
Supplement: Supplementary file 8 — Supplementary Software 1 [file 41467_2021_26213_MOESM8_ESM.zip › Supplementary Software 1/Analysis_Source_Code.pdf]

In [1]:
```
# import libraries
import os
import numpy as np
import pandas as pd
import matplotlib.pyplot as plt
import random as rand
```

In [2]:
```
setlen=lambda x:len(set(x))
def chgdir(dirname):
    """This wrapper creates a directory, if not already present, and then shifts code to that w
    if os.path.isdir(dirname):
        os.chdir(dirname)
        print('Changing directory to '+dirname)
    else:
        print('Directory '+dirname+' not found.\nCreating new directory.\nChanging to new direc
        os.mkdir(dirname)
        os.chdir(dirname)
```

In [3]:
```
cwd=os.getcwd()
cwd_input_matrices=os.path.join(cwd,'input_matrices')
cwd_interim=os.path.join(cwd,'interim_tmp_files')
cwd_genomics=os.path.join(cwd,'Genomics_Analysis')
cwd_SEER=os.path.join(cwd,'SEER_Analysis')
```

Import data from input_matrices

In [4]:
```
dfC=pd.read_csv(os.path.join(cwd_genomics,'Genomics_Output_Processed.txt'),sep='\t',index_col='
dfCred=dfC.drop(['All'],axis='columns').drop('Total') # genomic matrix without the total rows
dfCp=dfC.div(dfC.loc['Total':,:].values[0]) # P(gene|hist): Calculate probability of having a g
dfCp=(dfCp.drop(['All'], axis=1)).drop(['Total'])
```

In [5]:
```
# Calculate P(hist) from epidemiological studies
dfS0=pd.read_csv(os.path.join(cwd_SEER,'Output_SEER.txt'),sep='\t',index_col='HISTO CODE')
if (dfS0[-1:].values)[0,0] is np.nan:
    dfS0=dfS0[:-1] # remove the last (NAN) line.
dfS=dfS0.drop(index='CUSTOM SITES') # remove custom site names so histologies are only known by
dfS=dfS.astype('float') # convert all numbers to float

#P(hist AND loc): Calculate probability of incidence of a malignant cancer in a given location
sumStot=dfS.sum().sum()
dfSp=((dfS.transpose()).div(sumStot)).fillna(0.) # The only way the last row would become unity
dfSp=dfSp.astype('float')
```

In [6]:
```
# P(hist AND loc) including only ROSETTA histologies also incorporated in genomic data. For his
dfSC=dfS[dfCp.columns]
sumSCtot=dfSC.sum().sum()
dfSCp=((dfSC.transpose()).div(sumSCtot)).fillna(0.) # The only way the last row would become un
dfSCp=dfSCp.astype('float')
print(dfSCp.sum().sum()) # sum over histologies and locations for P(hist and tiss) =1
```

```
1.0000000000000002
```

In [7]:
```
dfp=dfCp@dfSCp # P(gene|loc)= sum over histologies in (p(gene|hist)*p(hist AND location))

dfNhist=pd.DataFrame({'Incidence':dfSC.sum(axis='index')}) # P(hist)= sum over locations for P(
dfSCNhist=dfCp.mul(dfNhist['Incidence'])# p(gene AND hist)=p(gene|hist)*p(hist) # probability r
dfSCNhist.columns=dfS0.loc['CUSTOM SITES'][dfSCNhist.columns]

dfIncd=pd.DataFrame({'Incidence':dfp.sum(axis='columns')}) # p(gene)=sum over locations on dfp
dfIncdsort=dfIncd.sort_values(by='Incidence',ascending=False)*100
```

Create In-Silico samples for genomics studies and find statistical measures

In [8]:
```
# Only genes above cut off percent incidence are considered for statistical analysis - This is
```

```
CutOffPercent=0.01 # select genes with incidence above a cut-off. Any percent level value cuts
NSilicoStudies=2000 # Select Number of silico studies ~ 2000 is enough to obtain stable central
dfIncdsort_sub=dfIncdsort[dfIncdsort>CutOffPercent].dropna()
print('Number of genes above cut-off incidence '+str(CutOffPercent)+'%:',len(dfIncdsort_sub),'\
```

```
Number of genes above cut-off incidence 0.01%: 20451
of total number of non-zero genes: 21271
```

Uncomment and run this line to skip de-novo generation of in-silico data below and speed processing along. df_Incdstats=pd.read_csv(os.path.join(cwd_interim,'df_Incdstats.csv'),sep='\t',index_col='Hugo_Symbol') df_Incdstats=df_Incdstats.astype(float)

In [ ]:

```
%%time
dfinp=(dfCred.copy(deep=True).loc[dfIncdsort_sub.index]).astype(int) # select genes with incide

df_SilicoStudies=dfinp.applymap(lambda x:np.random.poisson(x,NSilicoStudies) if x>0 else np.arr
print('Silico Studies Generated')
df_Silico_cases_ALL_Total=df_SilicoStudies+(dfC.drop(columns='All').loc['Total'].astype(int)-df
print('Denominator defined')
# For those rare cases where there are very few sequencing cases available.
dfAllboolean=df_Silico_cases_ALL_Total.applymap(lambda x:x<1).applymap(lambda x:any(x))
icollist=df_Silico_cases_ALL_Total.columns[dfAllboolean.any()]
indexlist=df_Silico_cases_ALL_Total.index[dfAllboolean.transpose().any()]
df_Silico_cases_ALL_Total.loc[indexlist,icollist]=df_Silico_cases_ALL_Total.loc[indexlist,icoll
print('Denominator Updated')
# Calculate conditional probability for silico samples
df_Silico_Cp=df_SilicoStudies.div(df_Silico_cases_ALL_Total) #divide the each case count by tot
print('Conditional Probabilities Generated')
del dfAllboolean
del df_SilicoStudies

df_Silico_p=df_Silico_Cp@dfSCp # Calcualte (p(gene|hist)*p(hist AND tissue)) for each in-silico
print('Total Probabilty Samples Generated')
del df_Silico_Cp
df_Silico_Incdsort=df_Silico_p.sum(axis='columns')*100
del df_Silico_p
print('Done.')
df_Incdstats=pd.DataFrame(df_Silico_Incdsort,columns=['Incidence_Silico'])
df_Incdstats['Incidence']=dfIncdsort.loc[df_Incdstats.index,'Incidence']
df_Incdstats['CL97.5%']=df_Incdstats['Incidence_Silico'].apply(lambda arr:np.percentile(arr,97.
df_Incdstats['CL2.5%']=df_Incdstats['Incidence_Silico'].apply(lambda arr:np.percentile(arr,2.5)
df_Incdstats['Mean']=df_Incdstats['Incidence_Silico'].apply(np.mean)
df_Incdstats=df_Incdstats[['Incidence','CL97.5%','CL2.5%','Mean']]
df_Incdstats.to_csv(os.path.join(cwd_interim,'df_Incdstats.csv'),sep='\t')
del df_Silico_Incdsort
```

Import list of genes in RAS pathway, Kinome, Consensus 1 and 2

In [9]:

```
dfGA=pd.read_csv(os.path.join(cwd_input_matrices,'List_Ras_Kinome.txt'),sep='\t') # annotated l
dfCT=pd.read_csv(os.path.join(cwd_input_matrices,'Lists_CT1and2.txt'),sep='\t') # Updated 2020
# remove NAN values and isolate the proteins for which length is available.
kinomelist=set([elem for elem in dfGA['Kinome'] if elem is not np.nan])
CT1list=set([elem for elem in dfCT['Consensus_Tier1'] if elem is not np.nan])
CT2list=set([elem for elem in dfCT['Consensus_Tier2'] if elem is not np.nan])
CT12list=CT1list.union(CT2list)
Raslist=set([elem for elem in dfGA['RAS_Pathway'] if elem is not np.nan])

def checkindices(l1):
    """Returns TRUE if all items present in dfIncdsort else returns a list of indices absent""'
    flag=True
    listfalse=[]
    for item in l1:
        flag=flag and (item in dfIncdsort.index)
        if not (item in dfIncdsort.index):
            listfalse=listfalse+[item]
    return flag if flag else listfalse
```

Import validation data from Kandoth et al and Bailey et al.

```
In [10]: dfTCGA13=pd.read_csv(os.path.join(cwd_input_matrices,'TCGA2013_Kandoth_Supplementary_Table_4.tx
         dfTCGA13.index=dfTCGA13.index.map(lambda x:x.upper())
         dfTCGA18=pd.read_csv(os.path.join(cwd_input_matrices,'TCGA_2018_Bailey.txt'),sep='\t',index_col
         dfTCGA18.index=dfTCGA18.index.map(lambda x:x.upper())
```

```
In [11]: #Import alternate gene nomenclature file from cbioportal: https://docs.cbioportal.org/3.-cbiopo
         #Homo_sapien.gene_info.gz ftp://ftp.ncbi.nih.gov/gene/DATA/GENE_INFO/Mammalia/Homo_sapiens.gene
         dfGeneNames=pd.read_csv(os.path.join(cwd_input_matrices,'Homo_sapiens_gene_info_GM.txt'),sep='\
         dfGeneNames=dfGeneNames.astype(str).applymap(lambda x:x.upper())
         dfGeneNames.Synonyms=[str(row).split('|') for row in dfGeneNames.Synonyms.values]
         listSynonyms=[elem for row in dfGeneNames.Synonyms.values for elem in row]
         def FindGeneName(igene):
             # This function has already been applied on the input gene list. In that application, care
             igene=igene.upper()
             retgene=igene
             if (igene in set(dfGeneNames.Symbol)) or (igene not in set(listSynonyms) or (igene in dfC.i
                 return retgene
             else:
                 retgene=dfGeneNames[[igene in row for row in dfGeneNames.Synonyms]].Symbol.values[0]
                 return retgene
```

```
In [12]: def chgdir(dirname):
             if os.path.isdir(dirname):
                 os.chdir(dirname)
                 print('Changing directory to '+dirname)
             else:
                 print('Directory '+dirname+' not found.\nCreating new directory.\nChanging to new direc
                 os.mkdir(dirname)
                 os.chdir(dirname)
         chgdir('Results')
```

```
Changing directory to Results
```

Figure 2A

```
In [14]: "Rows and columns in Genomics Matrix:",len(dfC.index)-1, len(dfC.columns)-1 # One extra "All" c
```

```
Out[14]: ('Rows and columns in Genomics Matrix:', 21271, 73)
```

```
In [15]: 'The number of samples sequenced in Genomics studies included:', dfC.loc['Total','All']
```

```
Out[15]: ('The number of samples sequenced in Genomics studies included:', 19181)
```

```
In [16]: 'Total number of cancer diagnoses included (SEER):',round(dfS.sum().sum(),2)
```

```
Out[16]: ('Total number of cancer diagnoses included (SEER):', 7167808.0)
```

```
In [17]: print('Total number of histologies included in ICD03 <or> ROSETTA:', len(dfS.sum()),'\nNumber c
```

```
Total number of histologies included in ICD03 <or> ROSETTA: 716
Number of histologies in ROSETTA with at least one case included: 370
```

```
In [18]: round(100*sumSCtot/sumStot,2) # Percentage of cases from SEER database which are included in ou
```

```
Out[18]: 92.95
```

Figure 2B

```
In [23]: # Replace Gene Names From Redundant gene names list
         print(len(dfTCGA13.index),len(set([FindGeneName(igene) for igene in dfTCGA13.index])))
         if len(dfTCGA13.index)==len(set([FindGeneName(igene) for igene in dfTCGA13.index])):
             dfTCGA13.index=[FindGeneName(igene) for igene in dfTCGA13.index]
```

```
127 127
```

```
In [24]: checkindices(dfTCGA13.index)
```

Out[24]: ['MALAT1', 'MIR142']

In [25]:
```
if checkindices(dfTCGA13.index)!=True: # check that all indices are present in our prevalance v
    dfTCGA13=dfTCGA13.drop(checkindices(dfTCGA13.index))
```

In [26]:
```
checkindices(dfTCGA13.index) # check that all indices are present in our prevalance vector
```

Out[26]: True

Figure 2D

In [27]:
```
dfTCGA13.drop('TP53',inplace=True)
```

In [28]:
```
font = {'family' : 'Arial',
        'weight' : 'normal',
        'size'   : 16}
plt.rc('font', **font)
plt.figure(figsize=(4,4))
dfIncdsortTCGA13genes=df_Incdstats.loc[dfTCGA13.index] # extract the TCGA genes from our preval
xvals=dfTCGA13['Pancan12 Freq%'].values
yvals=dfIncdsortTCGA13genes['Incidence'].values
yerr=[abs(dfIncdsortTCGA13genes['CL2.5%']-dfIncdsortTCGA13genes['Incidence']).values,(dfIncdsor
dfdiff=pd.concat([dfTCGA13[['Pancan12 Freq%']],dfIncdsortTCGA13genes[['Incidence','CL97.5%','CL
xyline=np.arange(0,20)
plt.plot(xyline,xyline,color='gray')
plt.errorbar(x=xvals,y=yvals,yerr=yerr,color='k',fmt='o',ms=6,elinewidth=3)
plt.xlabel('Proportion of TCGA Samples (%)\n (Kandoth 2013)',size=20)
plt.ylabel('Estimated Mutation Frequency\n in U.S. population (%)',size=20)
dfdiffsort=dfdiff.loc[dfdiff['abs(PrevDiff)']>3]
# dictextloc={'PIK3CA':1.9,'PTEN':1.5, 'VHL':1, 'KRAS':1.55, 'BRAF':1.45, 'KMT2D':1.85}
for id1 in dfdiffsort.index:
    plt.text(dfdiffsort['Pancan12 Freq%'][id1]+0.2,dfdiffsort['Incidence'][id1]+0.2,id1,fontsiz
plt.xlim([0,20])
plt.ylim([0,20])
plt.gca().spines['right'].set_linewidth(3)
plt.gca().spines['top'].set_linewidth(3)
plt.gca().spines['bottom'].set_linewidth(3)
plt.gca().spines['left'].set_linewidth(3)
plt.gca().xaxis.set_tick_params(width=3,size=10,labelsize=20)
plt.gca().yaxis.set_tick_params(width=3,size=10,labelsize=20)

fname='Figure2D_TCGApancanKandoth_scatter'
dfdiff.to_excel(fname+'.xlsx')
plt.savefig(fname+'.pdf',bbox_inches='tight')
plt.close() # replace with plt.show() to display chart
```

Figure 2E

In [29]:
```
# Replace Gene Names From Redundant gene names list
print(len(dfTCGA18.index),len(set([FindGeneName(igene) for igene in dfTCGA18.index])))
if len(dfTCGA18.index)==len(set([FindGeneName(igene) for igene in dfTCGA18.index])):
    dfTCGA18.index=[FindGeneName(igene) for igene in dfTCGA18.index]
```

299 299

In [30]:
```
checkindices(dfTCGA18.index) # check that all indices are present in our prevalance vector
```

Out[30]: True

In [31]:
```
dfTCGA18=dfTCGA18[dfTCGA18.sum(axis='columns')>0.]*100
```

In [32]:
```
dfTCGA18.drop('TP53',inplace=True)
```

In [ ]:
```
font = {'family' : 'Arial',
        'weight' : 'normal',
        'size'   : 16}
plt.rc('font', **font)
plt.figure(figsize=(4,4))
plt.rcParams['pdf.fonttype'] = 42 # To make output Adobe Illustrator friendly for formatting th
dfIncdsortTCGA18genes=df_Incdstats.loc[dfTCGA18.index] # extract the TCGA genes from our preval
xvals=dfTCGA18['Pancan Freq%'].values
yvals=dfIncdsortTCGA18genes['Incidence'].values
yerr=[abs(dfIncdsortTCGA18genes['CL2.5%']-dfIncdsortTCGA18genes['Incidence']).values,(dfIncdsor
dfdiff=pd.concat([dfTCGA18[['Pancan Freq%']],dfIncdsortTCGA18genes[['Incidence','CL97.5%','CL2.
xyline=np.arange(0,20)
plt.plot(xyline,xyline,color='gray')
plt.errorbar(x=xvals,y=yvals,yerr=yerr,color='k',fmt='o',ms=6,elinewidth=3)
plt.xlabel('Proportion of TCGA Samples (%)\n (Bailey 2018)',size=20)
plt.ylabel('Estimated Mutation Proportion\n in U.S. population (%)',size=20)
dfdiffsort=dfdiff.loc[dfdiff['abs(PrevDiff)']>3]
dictextloc={'PIK3CA':1.9,'PTEN':1.5, 'VHL':1, 'KRAS':0.2, 'BRAF':1.45, 'KMT2D':1.85,'APC':2.,'I
for id1 in dfdiffsort.index:
    plt.text(dfdiffsort['Pancan Freq%'][id1]-dictextloc[id1],dfdiffsort['Incidence'][id1]+0.5,i
plt.xlim([0,20])
plt.ylim([0,20])
plt.gca().spines['right'].set_linewidth(3)
plt.gca().spines['top'].set_linewidth(3)
plt.gca().spines['bottom'].set_linewidth(3)
plt.gca().spines['left'].set_linewidth(3)
plt.gca().xaxis.set_tick_params(width=3,size=10,labelsize=20)
plt.gca().yaxis.set_tick_params(width=3,size=10,labelsize=20)

fname='Figure2E_TCGApancanBailey_scatter'
dfdiff.to_excel(fname+'.xlsx')
plt.savefig(fname+'.pdf',bbox_inches='tight')
plt.close() # replace with plt.show() to display chart
```

Compare impact of epidemiological reweighting for our genomic dataset

In [34]:
```
%%time
# In-Silico Studies for Statistical Analysis
# Only genes above cut off percent incidence are considered for statistical analysis - This is
print('Number of genes above cut-off incidence '+str(CutOffPercent)+'%:',len(dfIncdsort_sub),'\

def fun_Silico_Unwtd(dflist):
    """Inputs a dataframe of numbers and creates Poisson in-silico studies"""
    import numpy as np
    inpdf=dflist[0]
    dfC_AllTot=dflist[1]
    NumSilicoStudies=dflist[2] # NUMBER of IN-SILICO SAMPLES generated for each gene and ROSETT
    df_SilicoStudies=inpdf.apply(lambda x:np.random.poisson(x,NumSilicoStudies))
    df_Silicodiff=df_SilicoStudies-inpdf
    df_Silico_cases_ALL_Total=df_Silicodiff+dfC_AllTot
    df_Silico_Cp=df_SilicoStudies.div(df_Silico_cases_ALL_Total)*100 #divide the each case coun
    del df_SilicoStudies
    del df_Silicodiff
    del df_Silico_cases_ALL_Total
    return df_Silico_Cp

#Process in-silico studies in parallel
dfinp=dfCred.sum(axis='columns').loc[dfIncdsort_sub.index] # select genes with incidence above
datalist=[dfinp,dfC.All.Total,NSilicoStudies]

df_Silico_Cp=fun_Silico_Unwtd(datalist)

# stats for unweighted genomic Incdalence rates
df_unwtd_Incdstats=pd.DataFrame(df_Silico_Cp,columns=['Incidence_Silico'])
df_unwtd_Incdstats['Mean']=df_unwtd_Incdstats['Incidence_Silico'].apply(np.mean)
df_unwtd_Incdstats['CL97.5%']=df_unwtd_Incdstats['Incidence_Silico'].apply(lambda arr:np.percer
df_unwtd_Incdstats['CL2.5%']=df_unwtd_Incdstats['Incidence_Silico'].apply(lambda arr:np.percent
df_unwtd_Incdstats[['Mean','CL97.5%','CL2.5%']].to_csv(os.path.join(cwd_interim,'df_unwtd_Incds
```

```
Number of genes above cut-off incidence 0.01%: 20451
```

```
of total number of non-zero genes: 21271
Wall time: 8.63 s
```

In [35]:
```
font = {'family' : 'Arial',
        'weight' : 'normal',
        'size'   : 20}
plt.rc('font', **font)
plt.rcParams['pdf.fonttype'] = 42 # To make output Adobe Illustrator friendly for formatting th

plt.figure(figsize=(4,4))
genelist_G=dfIncdsort_sub.index.values
df_xplt=df_unwtd_Incdstats.loc[genelist_G].copy(deep=True)
df_yplt=df_Incdstats.loc[genelist_G].copy(deep=True)
xvals=df_xplt.Mean.loc[genelist_G].values
yvals=df_yplt.Mean.loc[genelist_G].values
plt.scatter(x=xvals,y=yvals,marker='o',color='k',s=10)
xyline=np.arange(0,40)
plt.plot(xyline,xyline,color='gray')

genesublist_G=[row for row in genelist_G if df_xplt.Mean[row]>10 or df_yplt.Mean[row]>10]
xvals=df_xplt.Mean.loc[genesublist_G].values
yvals=df_yplt.Mean.loc[genesublist_G].values
xerror=[abs(df_xplt.loc[genesublist_G,'CL2.5%']-df_xplt.loc[genesublist_G,'Mean']).values,(df_x
yerror=[abs(df_yplt.loc[genesublist_G,'CL2.5%']-df_yplt.loc[genesublist_G,'Mean']).values,(df_y
plt.errorbar(x=xvals,y=yvals,xerr=xerror,yerr=yerror,color='k',fmt='o',ms=1,elinewidth=3)

labelgenes=[igene for igene in genelist_G if abs(df_xplt.Mean.loc[igene]-df_yplt.Mean.loc[igene
# dicloctexty={ 'TTN', 'MUC16', 'CSMD3', 'PIK3CA', 'LRP1B', 'RYR2', 'USH2A', 'ZFHX4', 'APC','TP
# dicloctextx={'FAT1':-4.5, 'KMT2D':0.2, 'LRP1B':1., 'KRAS':1, 'CDKN2A':0.4, 'TP53':0.7,'APC':0
for id1 in labelgenes:
    plt.text(x=df_xplt.loc[id1,'Mean']-7,y=df_yplt.loc[id1,'Mean']+1.5,s=id1,fontsize=16,color=
plt.text(x=36,y=33,s='x = y',fontsize=20,color='gray',rotation=30)
plt.xlim([0,40])
plt.ylim([0,40])
plt.gca().spines['right'].set_linewidth(2)
plt.gca().spines['top'].set_linewidth(2)
plt.gca().spines['bottom'].set_linewidth(2)
plt.gca().spines['left'].set_linewidth(2)
plt.gca().xaxis.set_tick_params(width=3,size=10,labelsize=20)
plt.gca().yaxis.set_tick_params(width=3,size=10,labelsize=20)

plt.xlabel('Unweighted Genomics Data Mutation Proportion (%)',size=20)
plt.ylabel('Weighted Mutation Proportion\n in U.S. Population (%)',size=20)
fname='Figure_GenomicsCompare_scatter'
plt.savefig(fname+'.pdf',bbox_inches='tight')
plt.close() # replace with plt.show() to display chart
```

In [36]:
```
df_xplt.columns=[icol+'_Unwtd' for icol in df_xplt.columns]
```

In [37]:
```
dfdiff=pd.concat([df_xplt[['Mean_Unwtd','CL97.5%_Unwtd','CL2.5%_Unwtd']],df_yplt[['Mean','CL97.
dfdiff.loc[dfdiff['abs(MeanDiff)']>0.5].loc[dfdiff['MeanDiffRatio(%)']>40].sort_values(by='Mear
```

Figure 3A

In [49]:
```
CT1list=[FindGeneName(igene) for igene in CT1list]
CT2list=[FindGeneName(igene) for igene in CT2list]
CT12list=[FindGeneName(igene) for igene in CT12list]
```

In [50]:
```
# identify Incidence rates by CT1, CT2 and CT12 lists , SORT and store as percentages
print(checkindices(CT1list),checkindices(CT2list),checkindices(CT12list))
CT2list=[item for item in CT2list if item not in checkindices(CT2list)]
CT12list=[item for item in CT12list if item not in checkindices(CT12list)]
```

```
True ['HMGN2P46', 'MALAT1'] ['MALAT1', 'HMGN2P46']
```

In [40]:
```
dfCT1prev=(dfIncd.loc[CT1list].sort_values(by=['Incidence'],ascending=False)*100)
dfCT1prev['CL97.5%']=[df_Incdstats.loc[igene,'CL97.5%'] if igene in df_Incdstats.index else np.
```

```
dfCT1prev['CL2.5%']=[df_Incdstats.loc[igene,'CL2.5%'] if igene in df_Incdstats.index else np.na
dfCT1prev['Mean_Silico']=[df_Incdstats.loc[igene,'Mean'] if igene in df_Incdstats.index else np
dfCT1prev.round(5).to_excel('Figure3_Incidences_CT1.xlsx')
```

In [41]:

```
dfCT2prev=(dfIncd.loc[CT2list].sort_values(by=['Incidence'],ascending=False)*100)
dfCT2prev['CL97.5%']=[df_Incdstats.loc[igene,'CL97.5%'] if igene in df_Incdstats.index else np.
dfCT2prev['CL2.5%']=[df_Incdstats.loc[igene,'CL2.5%'] if igene in df_Incdstats.index else np.na
dfCT2prev['Mean_Silico']=[df_Incdstats.loc[igene,'Mean'] if igene in df_Incdstats.index else np
dfCT2prev.round(5).to_excel('Figure3_Incidences_CT2.xlsx')
```

In [42]:

```
dfCT12prev=(dfIncd.loc[CT12list].sort_values(by=['Incidence'],ascending=False)*100)
dfCT12prev['CL97.5%']=[df_Incdstats.loc[igene,'CL97.5%'] if igene in df_Incdstats.index else np
dfCT12prev['CL2.5%']=[df_Incdstats.loc[igene,'CL2.5%'] if igene in df_Incdstats.index else np.n
dfCT12prev['Mean_Silico']=[df_Incdstats.loc[igene,'Mean'] if igene in df_Incdstats.index else n
dfCT12prev.round(5).to_excel('Figure3_Incidences_CT12.xlsx')
```

Figure3A. Bar Chart of top 50 CT1 genes

In [51]:

```
len(CT12list),setlen(CT12list)
```

Out[51]:

```
(714, 714)
```

In [44]:

```
axis_font = {'size':'10'}
dfbarplt=dfCT1prev[:50]
barlabels=dfbarplt.index.values
barpos=np.arange(len(barlabels))
bardata=dfbarplt['Incidence'].values
barerr=[abs(dfbarplt['CL2.5%']-dfbarplt['Incidence']).values,(dfbarplt['CL97.5%']-dfbarplt['Inc
figobj,axobj=plt.subplots(figsize=(15,10))
axobj.bar(barpos,bardata,yerr=barerr,align='center',alpha=0.5,edgecolor='k')
plt.xticks(barpos,barlabels,rotation=90,**axis_font)
plt.ylabel('% Incidence in US population')
plt.savefig('Figure3A_Top50_CT1.png',dpi=300,bbox_inches='tight')
plt.close() # replace with plt.show() to display chart
```

Figure 3B

In [52]:

```
setlen(kinomelist),len(kinomelist)
```

Out[52]:

```
(514, 514)
```

In [53]:

```
# identify Incidence rates by kinomelist, SORT and store as percentages and stats
kinomelist=[FindGeneName(igene) for igene in kinomelist]
print(checkindices(kinomelist))
kinomelist=[item for item in kinomelist if item not in checkindices(kinomelist)]
dfkinomeprev=(dfIncd.loc[kinomelist].sort_values(by=['Incidence'],ascending=False)*100).round(5
dfkinomeprev['CL97.5%']=[df_Incdstats.loc[igene,'CL97.5%'] if igene in df_Incdstats.index else
dfkinomeprev['CL2.5%']=[df_Incdstats.loc[igene,'CL2.5%'] if igene in df_Incdstats.index else np
dfkinomeprev['Mean_Silico']=[df_Incdstats.loc[igene,'Mean'] if igene in df_Incdstats.index else
dfkinomeprev.to_excel('Figure3B_Incidences_kinome.xlsx')
```

```
['CAMK1B', 'MAP3K14', 'CCRK', 'PRKY']
```

In [59]:

```
axis_font = {'size':'10'}
dfbarplt=dfkinomeprev[:50]
barlabels=dfbarplt.index.values
barpos=np.arange(len(barlabels))
bardata=dfbarplt['Incidence'].values
barerr=[abs(dfbarplt['CL2.5%']-dfbarplt['Incidence']).values,(dfbarplt['CL97.5%']-dfbarplt['Inc
figobj,axobj=plt.subplots(figsize=(15,10))
axobj.bar(barpos,bardata,yerr=barerr,align='center',alpha=0.5,color='g',edgecolor='k')
plt.xticks(barpos,barlabels,rotation=90,**axis_font)
plt.ylabel('% Incidence in US population')
plt.savefig('Figure3B_Top50_kinome.png',dpi=150,bbox_inches='tight')
plt.close() # replace with plt.show() to display chart
```

Figure 3C

In [61]:
```
# identify Incidence rates by raslist, SORT and store as percentages and stats
Raslist=[FindGeneName(igene) for igene in Raslist]
print(checkindices(Raslist))
Raslist=[item for item in Raslist if item not in checkindices(Raslist)]
dfrasprev=(dfIncd.loc[Raslist].sort_values(by=['Incidence'],ascending=False)*100).round(5)
dfrasprev['CL97.5%']=[df_Incdstats.loc[igene,'CL97.5%'] if igene in df_Incdstats.index else np.
dfrasprev['CL2.5%']=[df_Incdstats.loc[igene,'CL2.5%'] if igene in df_Incdstats.index else np.na
dfrasprev['Mean_Silico']=[df_Incdstats.loc[igene,'Mean'] if igene in df_Incdstats.index else np
dfrasprev.to_excel('Figure3C_Incidences_RasPathway.xlsx')
```

```
['RASSF10']
```

In [65]:
```
setlen(dfrasprev.index.values),len(dfrasprev.index.values)
```

Out[65]:
```
(226, 226)
```

In [66]:
```
axis_font = {'size':'10'}
dfbarplt=dfrasprev[:50]
barlabels=dfbarplt.index.values
barpos=np.arange(len(barlabels))
bardata=dfbarplt['Incidence'].values
barerr=[abs(dfbarplt['CL2.5%']-dfbarplt['Incidence']).values,(dfbarplt['CL97.5%']-dfbarplt['Inc
figobj,axobj=plt.subplots(figsize=(15,10))
axobj.bar(barpos,bardata,yerr=barerr,align='center',alpha=0.5,color='r',edgecolor='k')
plt.xticks(barpos,barlabels,rotation=90,**axis_font)
plt.ylabel('% Incidence in US population')
plt.savefig('Figure3C_Top50_ras.png',dpi=150,bbox_inches='tight')
plt.close() # replace with plt.show() to display chart
```

Table 1

In [67]:
```
# Define Adenocarcinoma histologies by code
adenocarclist=['81603','83173','83703','70117','70267','70397','70187','70197','70277','70287',
print('Number of adenocarcinomas in SEER epidemiological data:',dfS[adenocarclist].sum().sum(),
aclist=[val for val in adenocarclist if val in dfCred.columns]
print('Adenocarcinoma ROSETTA histologies NOT in current genomic data:',[[val+': '+dfS0.loc['CL
```

```
Number of adenocarcinomas in SEER epidemiological data: 4676878.54917131
Percent of SEER histologies which are adenocarcinoma: 65.2483792698034
Adenocarcinoma ROSETTA histologies NOT in current genomic data: [['70407: Other Adenocarcinom
a']]
```

In [68]:
```
histsredAC=aclist
dfCp_ac=dfCp[histsredAC] # genomic conditional probablity matrix without All genes and total hi
# P(hist AND loc) including only histologies also incorporated in genomic data. For histologies
sumSCtot_ac=dfSC[histsredAC].sum().sum()
dfSCp_ac=dfSCp.loc[histsredAC]
dfSCp_ac=(dfSCp_ac.div(sumSCtot_ac)).mul(sumSCtot) # Multiply by total cases over all histologi
print(dfSCp_ac.sum().sum()) #Cross-check: sum over histologies and tissues for P(hist and tiss)
dfp_ac=dfCp_ac@dfSCp_ac # sum over histologies for (p(gene|hist)*p(hist AND tissue))

# dfpG=dfC.drop()

# N(hist)
dfNloc_ac=pd.DataFrame({'Incidence':dfSC[histsredAC].sum(axis='columns')})
dfIncd_ac=pd.DataFrame({'Incidence':dfp_ac.sum(axis='columns')}) # p(mut)=sum over locations or
dfIncdsort_ac=dfIncd_ac.sort_values(by='Incidence',ascending=False)*100
```

```
1.0
```

In [69]:
```
%%time
# Uncomment and run when creating Table 1. (In jupyter, the key 'y' uncomments the whole block.
dfSCredNorm=dfSCNhist.loc[CT1list].div(dfSCNhist.loc[CT1list].sum(axis=1),axis='index')*100 # H
commonhistdic={}
for id1 in dfSCredNorm.index:
    tophistset=dfSCredNorm.loc[id1].sort_values(ascending=False)
```

```
    tophistset=tophistset.sort_values(ascending=False)[:3]
    tophiststr=[id2+'('+str(tophistset[id2].round(1))+'%) ' for id2 in tophistset.index] # repl
    commonhistdic[id1]=''.join(tophiststr)
df_commonhist=pd.DataFrame.from_dict(commonhistdic, orient='index',columns=['Common Cancers'])
# Run to calculate Table 1
dfIncdsort_ac.columns=['Incidence_AdenoCarcinomas']
df_Incid_GlobalvsAdeno=pd.concat([dfIncdsort.loc[CT1list].round(1),dfIncdsort_ac.loc[CT1list].r
df_Incid_GlobalvsAdeno.to_excel('Table1_ConsensusGenesFreq.xlsx',index_label='Gene')
```

```
Wall time: 441 ms
```

Figure 4A

In [70]:
```
squamouslist=dfS0.columns[dfS0.columns>='70207']
squamouslist=squamouslist[squamouslist<='70257']
sqlist=[elem for elem in squamouslist if elem in dfCred.columns] # extract those squamous ROSET

Melanomalist=['70097','70107','93633','87443']
Melanomalist=[elem for elem in Melanomalist if elem in dfCred.columns]# extract those melanoma

TCClist=['70167'] # To analyze any other single histology - this is an easy trick. enter histol
TCClist=[elem for elem in TCClist if elem in dfCred.columns]
```

In [71]:
```
TypeList=[aclist,squamouslist,Melanomalist,TCClist]
print("Percent of all cancers in epidemiological studies that are within major sub-classificati
```

```
Percent of all cancers in epidemiological studies that are within major sub-classifications of
adenocarcinoma, squamous cell carcinoma, melanoma, or transitional cell carcinoma:
 [64.0, 9.0, 5.0, 5.0]
```

Figure 4B,C

In [72]:
```
# A few categories are manually combined in the manuscript figure.
```

In [73]:
```
dfbar=dfNloc_ac.sort_values(by='Incidence',ascending=False)
dfbar=100*dfbar/dfbar.sum()
axis_font = {'size':'10'}
barlabels=dfbar[:20].index.values
barpos=np.arange(len(barlabels))
bardata=dfbar[:20]['Incidence'].values
figobj,axobj=plt.subplots(figsize=(10,10))
axobj.bar(barpos,bardata,align='center',alpha=0.5)
plt.xticks(barpos,barlabels,rotation=90,**axis_font)
plt.ylabel('Percent Incidence')
dfbar.to_excel('Figure4B_Adenocarcinoma_Types.xlsx')
plt.close() # replace with plt.show() to display chart
```

In [74]:
```
histsredSQ=sqlist
dfCp_sq=dfCp[histsredSQ] # genomic conditional probablity matrix without All genes and total hi
# P(hist AND loc) including only histologies also incorporated in genomic data. For histologies
sumSCtot_sq=dfSC[histsredSQ].sum().sum()
dfSCp_sq=dfSCp.loc[histsredSQ]
dfSCp_sq=(dfSCp_sq.div(sumSCtot_sq)).mul(sumSCtot) # Multiply by total cases over all histologi
print(dfSCp_sq.sum().sum()) #Cross-check: sum over histologies and tissues for P(hist and tiss)
dfp_sq=dfCp_sq@dfSCp_sq # sum over histologies for (p(gene|hist)*p(hist AND tissue))

# dfpG=dfC.drop()

# N(hist)
dfNloc_sq=pd.DataFrame({'Incidence':dfSC[histsredSQ].sum(axis='columns')})
dfIncd_sq=pd.DataFrame({'Incidence':dfp_sq.sum(axis='columns')}) # p(mut)=sum over locations or
dfIncdsort_sq=dfIncd_sq.sort_values(by='Incidence',ascending=False)*100
```

```
0.9999999999999998
```

In [75]:
```
dfbar=dfNloc_sq.sort_values(by='Incidence',ascending=False)
dfbar=100*dfbar/dfbar.sum()
axis_font = {'size':'10'}
```

```
barlabels=dfbar[:10].index.values
barpos=np.arange(len(barlabels))
bardata=dfbar[:10]['Incidence'].values
figobj,axobj=plt.subplots(figsize=(10,10))
axobj.bar(barpos,bardata,align='center',alpha=0.5)
plt.xticks(barpos,barlabels,rotation=90,**axis_font)
plt.ylabel('Percent Incidence')
dfbar.to_excel('Figure4C_SCC_Types.xlsx')
plt.close() # replace with plt.show() to display chart
```

Figure 4D,E,G,H

In [76]:

```
def genIncdsrtdat(histinplist,igenelist):
    """Output: Dataframe which includes Silico_Incidence_mean,  CL97.5, CL2.5\n Input:List of s
    histsred=histinplist # use aclist for adenocarcinomas and sqlist for squamous cell carcinom
    dfCp1=dfCp.loc[igenelist,histsred] # genomic conditional probablity matrix without All gene
    # P(hist AND loc) including only histologies also incorporated in genomic data. For histolo
    sumSCtot1=dfSC[histsred].sum().sum()
    dfSCp1=dfSCp.loc[histsred]
    dfSCp1=(dfSCp1/sumSCtot1)*sumSCtot # Multiply by total cases over all histologies and divid
    dfp1=dfCp1@dfSCp1 # sum over histologies for (p(gene|hist)*p(hist AND tissue))

    dfIncd1=pd.DataFrame({'Incidence':dfp1.sum(axis='columns')}) # p(mut)=sum over locations or
    dfIncdsort1=dfIncd1.sort_values(by='Incidence',ascending=False)*100

    dfinp=dfCred.loc[igenelist,histsred].astype(int)#.loc[dfIncdsort_sub1.index,histsred].astyp

    # Create silico study samples
    df_SilicoStudies1=dfinp.applymap(lambda x:np.random.poisson(x,NSilicoStudies))
    df_Silico_cases_ALL_Total=df_SilicoStudies1+(dfC.loc['Total',histsred].astype(int)-dfinp)

    # For those rare cases where there are very few sequencing cases available.
    dfAllboolean=df_Silico_cases_ALL_Total.applymap(lambda x:x<1).applymap(lambda x:any(x))
    icollist=df_Silico_cases_ALL_Total.columns[dfAllboolean.any()]
    indexlist=df_Silico_cases_ALL_Total.index[dfAllboolean.transpose().any()]
    df_Silico_cases_ALL_Total.loc[indexlist,icollist]=df_Silico_cases_ALL_Total.loc[indexlist,i

    # Calculate conditional probability for silico samples
    df_Silico_Cp1=df_SilicoStudies1.div(df_Silico_cases_ALL_Total) #divide the each case count
    del df_Silico_cases_ALL_Total
    del df_SilicoStudies1

    df_Silico_p1=df_Silico_Cp1@dfSCp1 # Calcualte (p(gene|hist)*p(hist AND tissue)) for each in
    del df_Silico_Cp1

    df_Silico_Incdsort1=df_Silico_p1.sum(axis='columns')*100
    del df_Silico_p1

    df_Incdstats1=pd.DataFrame(df_Silico_Incdsort1,columns=['Incidence_Silico'])
    df_Incdstats1['Incidence']=dfIncdsort1.loc[df_Incdstats1.index,'Incidence']
    df_Incdstats1['CL97.5%']=df_Incdstats1['Incidence_Silico'].apply(lambda arr:np.percentile(a
    df_Incdstats1['CL2.5%']=df_Incdstats1['Incidence_Silico'].apply(lambda arr:np.percentile(ar
    df_Incdstats1['Mean_Silico']=df_Incdstats1['Incidence_Silico'].apply(np.mean)
    df_Incdstats1=df_Incdstats1.drop(columns='Incidence_Silico')
    df_Incdstats1=df_Incdstats1.sort_values(by='Incidence',ascending=False)
    del df_Silico_Incdsort1

    return df_Incdstats1
```

In [77]:

```
Fig4histdic={'D':['Adenocarcinoma',aclist],'E':['Squamous Cell Carcinoma',sqlist],'G':['Melanom
```

In [78]:

```
%%time
for figlabel in ['D','E','G','H']: # Change this label to D,E,G,H to generate respective plots
    dfIncd_hist=genIncdsrtdat(Fig4histdic[figlabel][1],CT1list)
#    dfIncd_hist=dfIncd_hist.loc[[igene for igene in CT1list if igene in dfIncd_hist.index]]
    print('Done: ',Fig4histdic[figlabel][0])
    dfIncd_hist=dfIncd_hist.sort_values(by='Incidence',ascending=False)
```

```
    dfbarplt=dfIncd_hist[:25]
    axis_font = {'size':'10'}
    barlabels=dfbarplt.index.values
    barpos=np.arange(len(barlabels))
    bardata=dfbarplt['Incidence'].values
    barerr=[abs(dfbarplt['CL2.5%']-dfbarplt['Incidence']).values,(dfbarplt['CL97.5%']-dfbarplt[
    figobj,axobj=plt.subplots(figsize=(10,10))
    axobj.bar(barpos,bardata,yerr=barerr,align='center',alpha=0.5)
    plt.xticks(barpos,barlabels,rotation=90,**axis_font)
    plt.ylabel('Mutation Frequency in '+Fig4histdic[figlabel][0]+' (%)')
    figname='Figure4'+figlabel+'_Top25_'+Fig4histdic[figlabel][0]
    dfbarplt.to_excel(figname+'.xlsx')
    ST4='SuppTable4_'+Fig4histdic[figlabel][0]
    dfIncd_hist.to_excel(ST4+'.xlsx')
    plt.savefig(figname+'.png',dpi=300,bbox_inches='tight')
    plt.close()
```

```
Done:  Adenocarcinoma
Done:  Squamous Cell Carcinoma
Done:  Melanoma
Done:  Transitional Cell Carcinoma
Wall time: 10.7 s
```

Figure 4F

In [79]:
```
# Difference between two poisson distribution is a skellam distribution. However, each gene is
```

In [80]:
```
%%time
df_Incdstats_ac=pd.read_excel('SuppTable4_Adenocarcinoma.xlsx',index_col='Hugo_Symbol')
df_Incdstats_sq=pd.read_excel('SuppTable4_Squamous Cell Carcinoma.xlsx',index_col='Hugo_Symbol'
```

```
Wall time: 133 ms
```

In [81]:
```
genelist_CT1acsq=[igene for igene in CT1list if ((igene in df_Incdstats_ac.index) and (igene in
genelist_acsq=list(df_Incdstats_ac.index[df_Incdstats_ac.index.isin(df_Incdstats_sq.index)])
```

In [82]:
```
def nonoverlapping(igene):
    """returns True if adeno and squamous 95% cl are non-overlapping for input gene"""
    acmin=df_Incdstats_ac.loc[igene,'CL2.5%']
    sqmin=df_Incdstats_sq.loc[igene,'CL2.5%']
    acmax=df_Incdstats_ac.loc[igene,'CL97.5%']
    sqmax=df_Incdstats_sq.loc[igene,'CL97.5%']
    if (acmin>sqmax) or (sqmin>acmax):
        return True
    else:
        return False
```

In [83]:
```
SigGenes_AcvsSq_CT1=[igene for igene in genelist_CT1acsq if nonoverlapping(igene)]
len(SigGenes_AcvsSq_CT1),len(genelist_CT1acsq)
```

Out[83]:
```
(87, 570)
```

In [84]:
```
font = {'family' : 'Arial',
        'weight' : 'normal',
        'size'   : 20}
plt.rc('font', **font)
plt.rcParams['pdf.fonttype'] = 42 # To make output Adobe Illustrator friendly for formatting th

plt.figure(figsize=(4,4))
df_xplt=df_Incdstats_ac.copy()
df_yplt=df_Incdstats_sq.copy()

xvals=df_xplt.Incidence.loc[genelist_CT1acsq].values
yvals=df_yplt.Incidence.loc[genelist_CT1acsq].values
plt.scatter(x=xvals,y=yvals,marker='o',color='k',s=50)
xyline=np.arange(0,40)
plt.plot(xyline,xyline,color='gray')
```

```
genesublist_CT1acsq=[row for row in SigGenes_AcvsSq_CT1 if df_Incdstats_ac.Incidence[row]>6 or
xvals=df_xplt.Incidence.loc[genesublist_CT1acsq].values
yvals=df_yplt.Incidence.loc[genesublist_CT1acsq].values
xerror=[abs(df_xplt.loc[genesublist_CT1acsq,'CL2.5%']-df_xplt.loc[genesublist_CT1acsq,'Incidenc
yerror=[abs(df_yplt.loc[genesublist_CT1acsq,'CL2.5%']-df_yplt.loc[genesublist_CT1acsq,'Incidenc
plt.errorbar(x=xvals,y=yvals,xerr=xerror,yerr=yerror,color='k',fmt='o',ms=8,elinewidth=2)

labelgenes=[igene for igene in SigGenes_AcvsSq_CT1 if abs(df_xplt.Incidence.loc[igene]-df_yplt.
dicloctexty={'FAT1':0.4, 'KMT2D':0.5, 'LRP1B':0.3, 'KRAS':0.3, 'CDKN2A':-0.4, 'TP53':0.4,'APC':
dicloctextx={'FAT1':-4.5, 'KMT2D':0.2, 'LRP1B':1., 'KRAS':1, 'CDKN2A':0.4, 'TP53':0.7,'APC':0.2
for id1 in labelgenes:
    if id1 not in ['NFE2L2','CDKN2A','FAT1']:
        plt.text(x=df_xplt.loc[id1,'Incidence']+dicloctextx[id1],y=df_yplt.loc[id1,'Incidence']
# plt.text(x=36,y=33,s='x = y',fontsize=20,color='gray',rotation=30)
plt.xlim([0,40])
plt.ylim([0,70])
plt.gca().spines['right'].set_linewidth(3)
plt.gca().spines['top'].set_linewidth(3)
plt.gca().spines['bottom'].set_linewidth(3)
plt.gca().spines['left'].set_linewidth(3)
plt.gca().xaxis.set_tick_params(width=3,size=10,labelsize=20)
plt.gca().yaxis.set_tick_params(width=3,size=10,labelsize=20)

plt.xlabel('Adenocarcinoma (%)',size=20)
plt.ylabel('Squamous Cell Carcinoma (%)',size=20)
fname='Figure4F_SCCvsAdeno_scatter_CT1'
plt.savefig(fname+'.pdf',bbox_inches='tight')
plt.close() # replace with plt.show() to display chart
```

In [85]:

```
# How many genes have more incidence in SCC vs Adenocarcinoma?
[[igene,round(df_xplt.Incidence.loc[igene],2),round(df_yplt.Incidence.loc[igene],2)] for igene
```

Out[85]:

```
[['NOTCH1', 2.9, 10.43],
 ['LRP1B', 10.46, 23.12],
 ['CDKN2A', 1.71, 12.49],
 ['KMT2D', 6.86, 16.47],
 ['FAT1', 5.35, 14.71],
 ['NFE2L2', 1.2, 9.85],
 ['TP53', 33.7, 62.94]]
```

In [86]:

```
df_Incdstats_ac.loc[genelist_CT1acsq].to_excel(fname+'AC.xlsx')
df_Incdstats_sq.loc[genelist_CT1acsq].to_excel(fname+'SQ.xlsx')
```

Figure 4I

In [87]:

```
df_Incdstats_mel=pd.read_excel('SuppTable4_Melanoma.xlsx',index_col='Hugo_Symbol')
df_Incdstats_tcc=pd.read_excel('SuppTable4_Transitional Cell Carcinoma.xlsx',index_col='Hugo_Sy
```

In [88]:

```
df_RASstats=round(pd.concat([df_Incdstats_ac.loc[['KRAS','NRAS','HRAS']].rename(columns={'Incic
df_RASstats['Hist_Type']=['AC']*3+['SCC']*3+['M']*3+['TCC']*3
df_RAS=pd.DataFrame(index=['AC','SCC','M','TCC'],columns=['KRAS','NRAS','HRAS'])
for icol in df_RAS.columns:
    for idx in df_RAS.index:
        df_RAS.loc[idx,icol]=df_RASstats[df_RASstats.Hist_Type==idx].loc[icol,['Incidence','CL9
df_RAS.loc[['TCC','M','SCC','AC']]
```

Out[88]:

	KRAS	NRAS	HRAS
**TCC**	[4.252, 6.01, 2.763]	[2.041, 3.193, 1.031]	[6.122, 8.0, 4.333]
**M**	[1.394, 2.317, 0.589]	[24.272, 26.908, 21.523]	[1.889, 2.936, 0.954]
**SCC**	[1.101, 1.708, 0.564]	[0.753, 1.266, 0.316]	[3.212, 4.145, 2.353]
**AC**	[13.978, 14.486, 13.44]	[1.488, 1.732, 1.251]	[0.595, 0.76, 0.438]

Supplementary Tables

```
In [89]: chgdir('Supp_Tables/')
```

```
Directory Supp_Tables/ not found.
Creating new directory.
Changing to new directory.
```

```
In [94]: setlen(df_Incdstats.index),len(df_Incdstats.index)
```

```
Out[94]: (20451, 20451)
```

```
In [90]: dfHistIncd=dfSp.sum(axis='columns') #p(hist) from SEER data
         dfHistIncd=dfHistIncd.sort_values(ascending=False)*100
         dfHPsorted=pd.DataFrame(columns=['CUSTOM SITES','Incidence'],index=dfHistIncd.index)
         dfHPsorted['CUSTOM SITES']=dfS0.loc['CUSTOM SITES']
         dfHPsorted['Incidence']=dfHistIncd
         dfHPsorted.to_excel('SuppTable1_ROSETTA_Abundance.xlsx')
```

```
In [91]: df_Incdstats.sort_values(by='Incidence',ascending=False).round(5).to_excel('SuppTableIII_AllGen
```

Supp Table III is a combination of Figure3_ CT1, RAS, Kinome and AllGenes txt files already output to the Results folder. # Supp Table IV is a combination of four histological incidence txt files already output to the Results folder with names starting with- SuppTable4_.
