# Supplementary material for: Cancer gene mutation frequencies for the U.S. population
Source: Nat Commun. 2021 Oct 13;12:5961. doi: 10.1038/s41467-021-26213-y (PMC8514428; doi:10.1038/s41467-021-26213-y)
Supplement: Supplementary file 8 — Supplementary Software 1 [file 41467_2021_26213_MOESM8_ESM.zip › Supplementary Software 1/Results/Figure_GenomicsCompare_scatter.pdf]

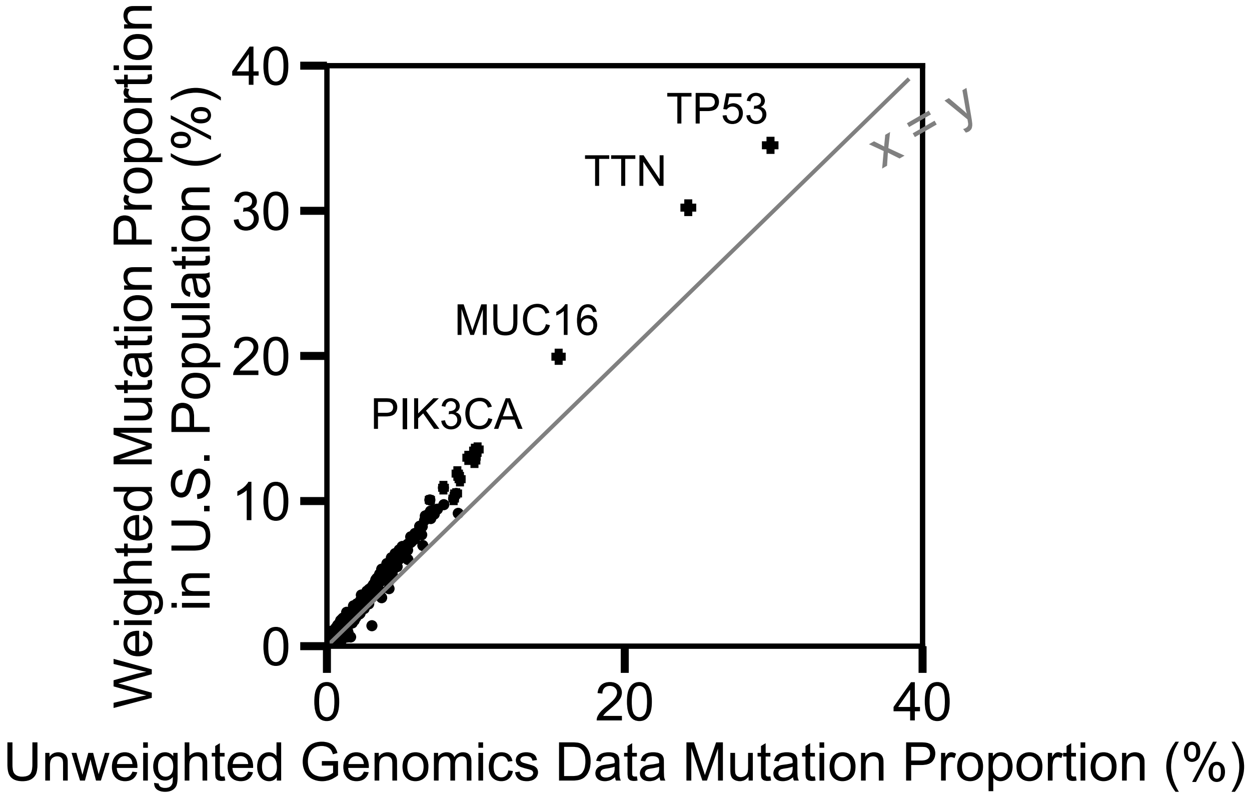

Weighted Mutation Proportion in U.S. Population (%)
40
30
20
10
0
0
20
40
Unweighted Genomics Data Mutation Proportion (%)
TP53
TTN
MUC16
PIK3CA
x = y
