# Supplementary figures and images for: Cancer gene mutation frequencies for the U.S. population
Source: Nat Commun. 2021 Oct 13;12:5961. doi: 10.1038/s41467-021-26213-y (PMC8514428; doi:10.1038/s41467-021-26213-y)

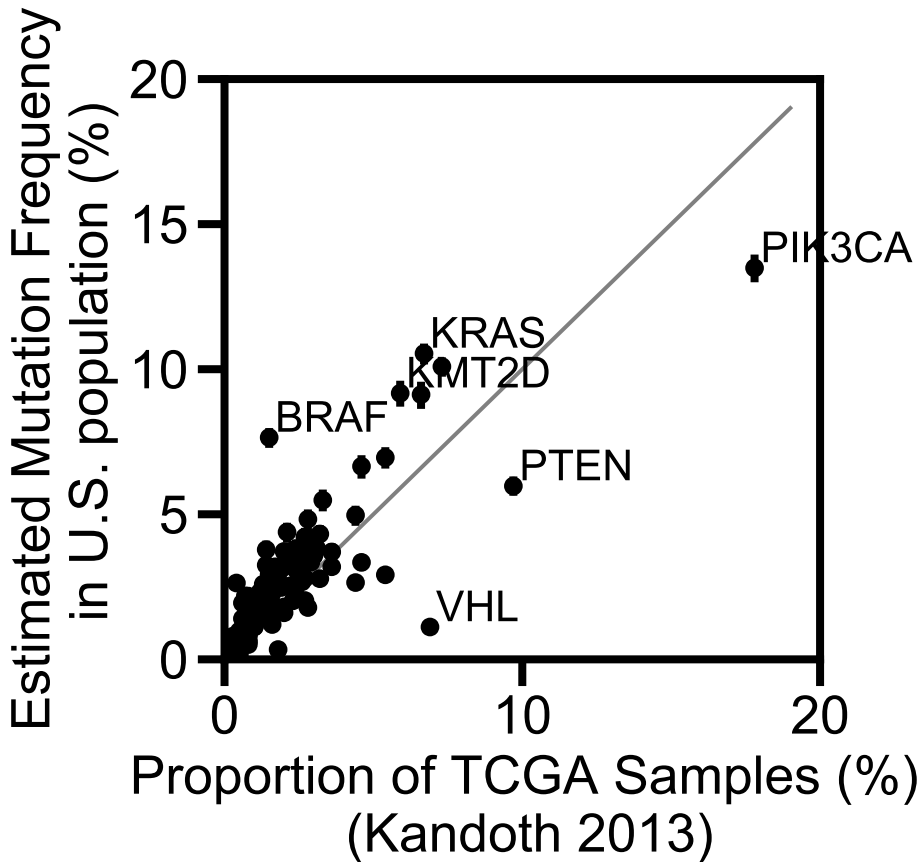

Supplement: Supplementary file 8 — Supplementary Software 1 [file 41467_2021_26213_MOESM8_ESM.zip › Supplementary Software 1/Results/Figure2D_TCGApancanKandoth_scatter.pdf]

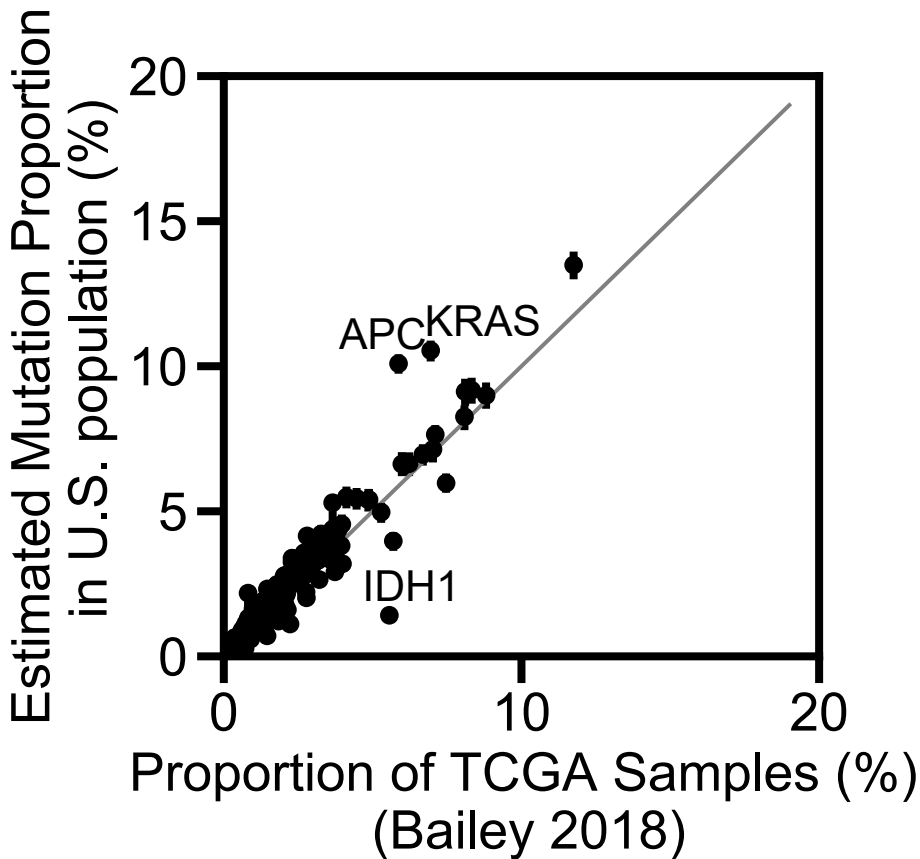

Supplement: Supplementary file 8 — Supplementary Software 1 [file 41467_2021_26213_MOESM8_ESM.zip › Supplementary Software 1/Results/Figure2E_TCGApancanBailey_scatter.pdf]

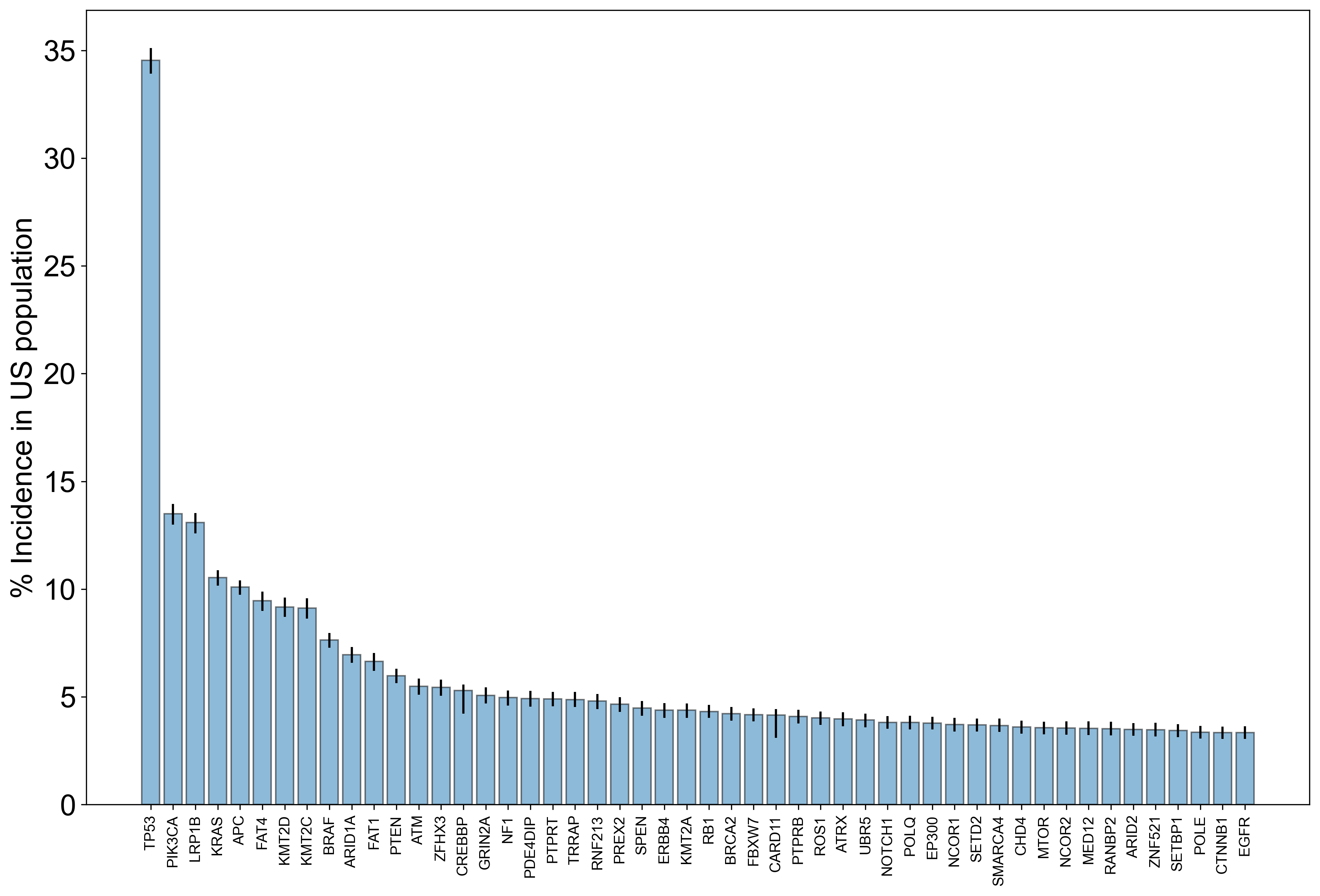

Supplement: Supplementary file 8 — Supplementary Software 1 [file 41467_2021_26213_MOESM8_ESM.zip › Supplementary Software 1/Results/Figure3A_Top50_CT1.png]

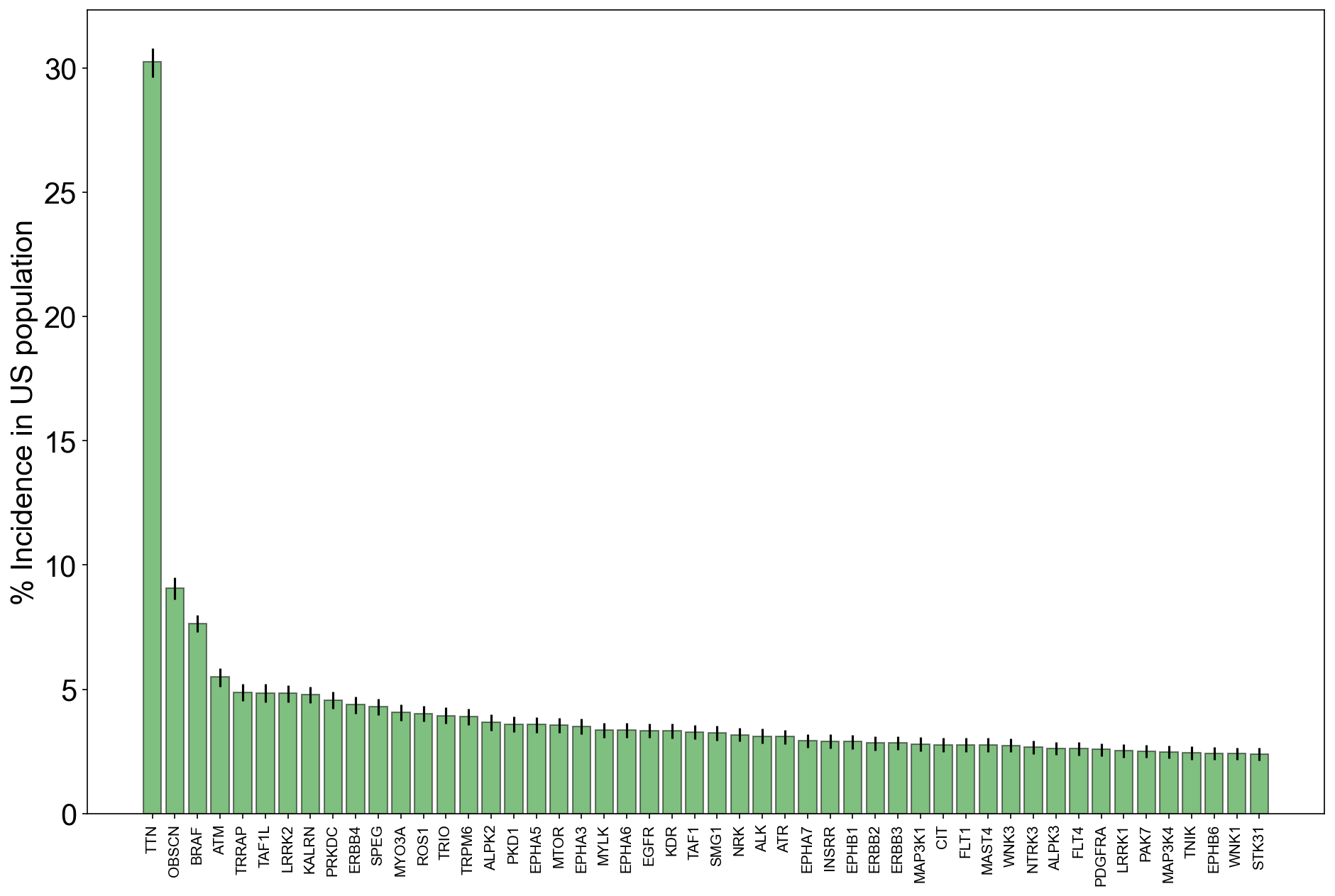

Supplement: Supplementary file 8 — Supplementary Software 1 [file 41467_2021_26213_MOESM8_ESM.zip › Supplementary Software 1/Results/Figure3B_Top50_kinome.png]

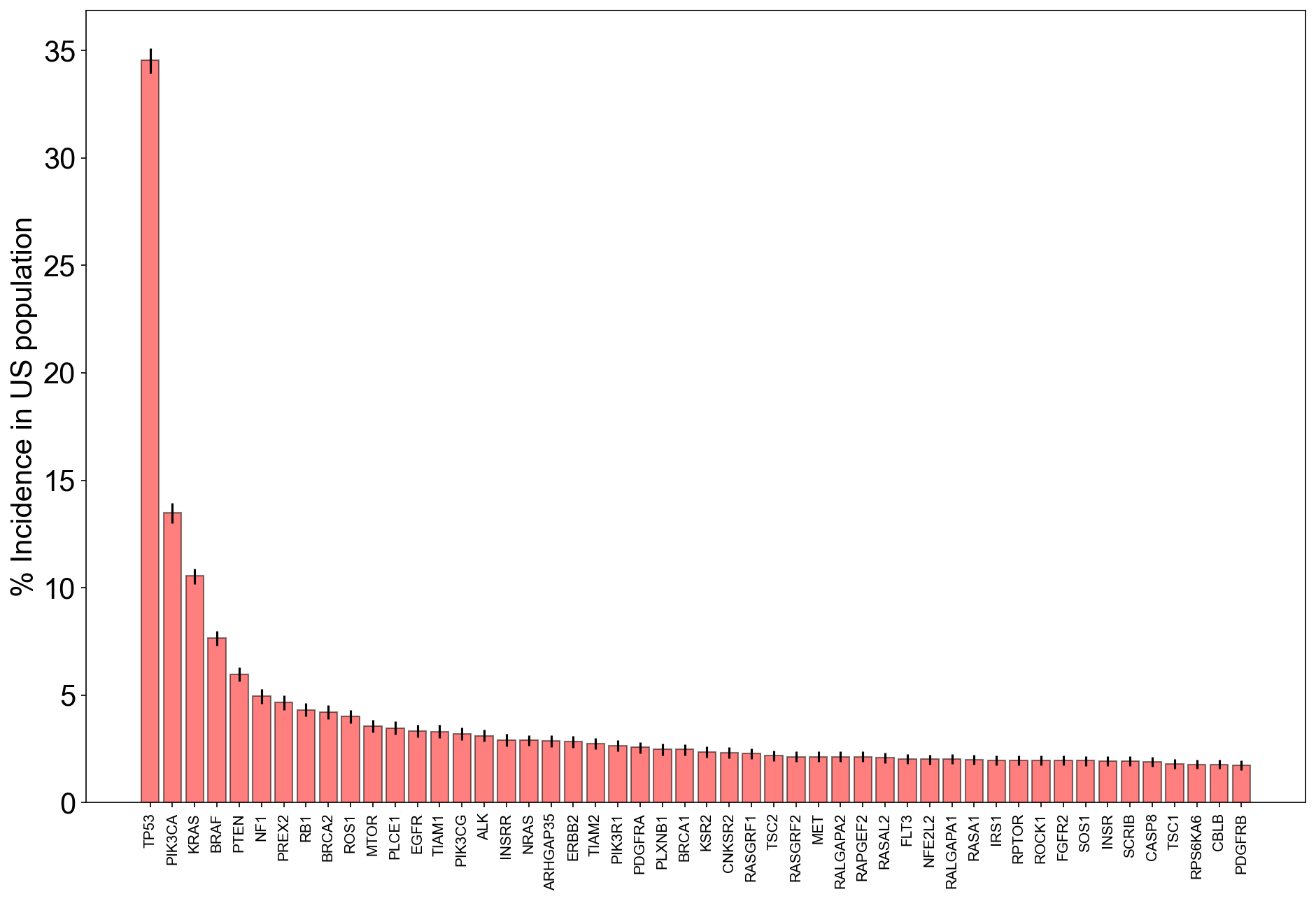

Supplement: Supplementary file 8 — Supplementary Software 1 [file 41467_2021_26213_MOESM8_ESM.zip › Supplementary Software 1/Results/Figure3C_Top50_ras.png]

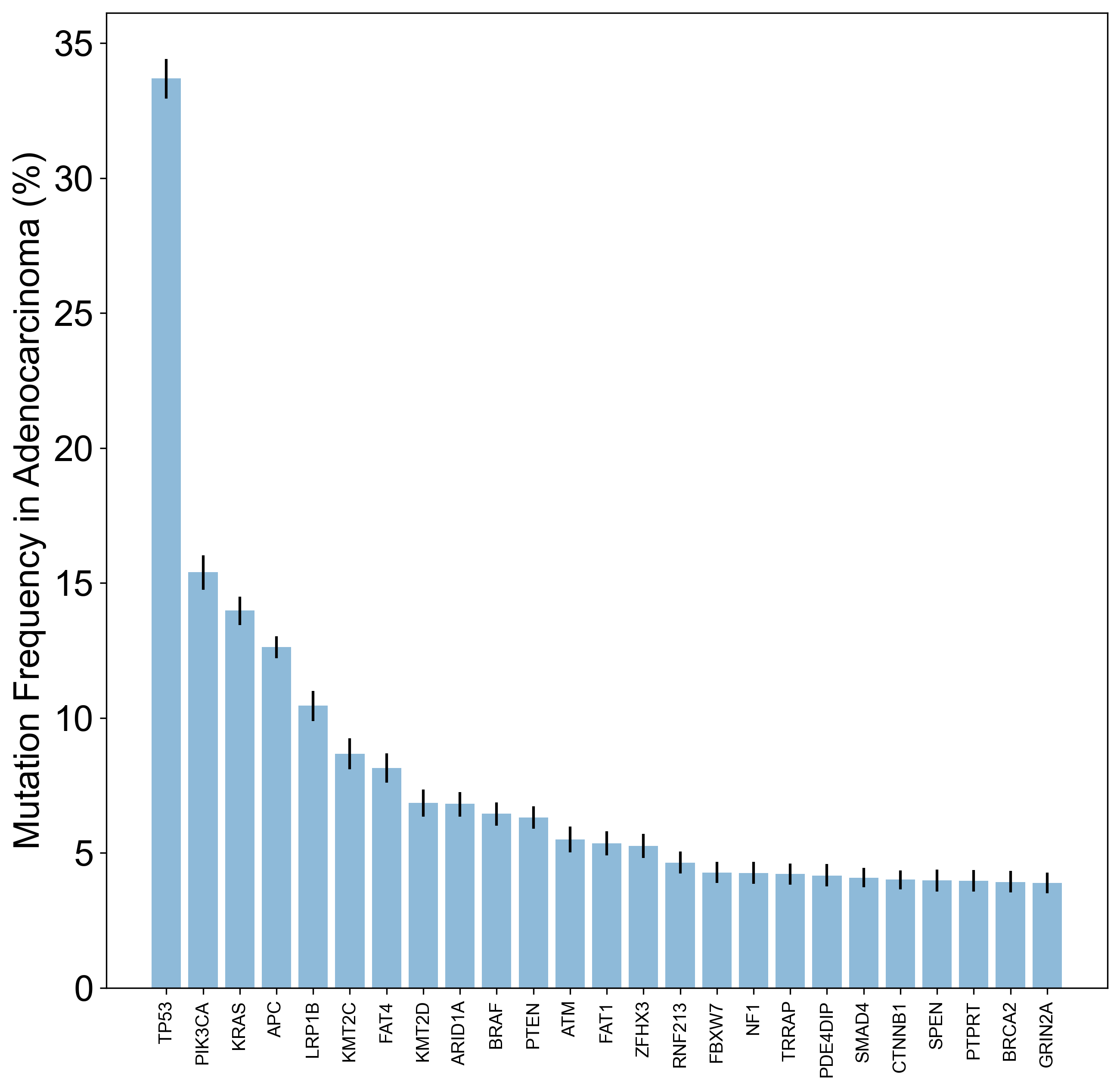

Supplement: Supplementary file 8 — Supplementary Software 1 [file 41467_2021_26213_MOESM8_ESM.zip › Supplementary Software 1/Results/Figure4D_Top25_Adenocarcinoma.png]

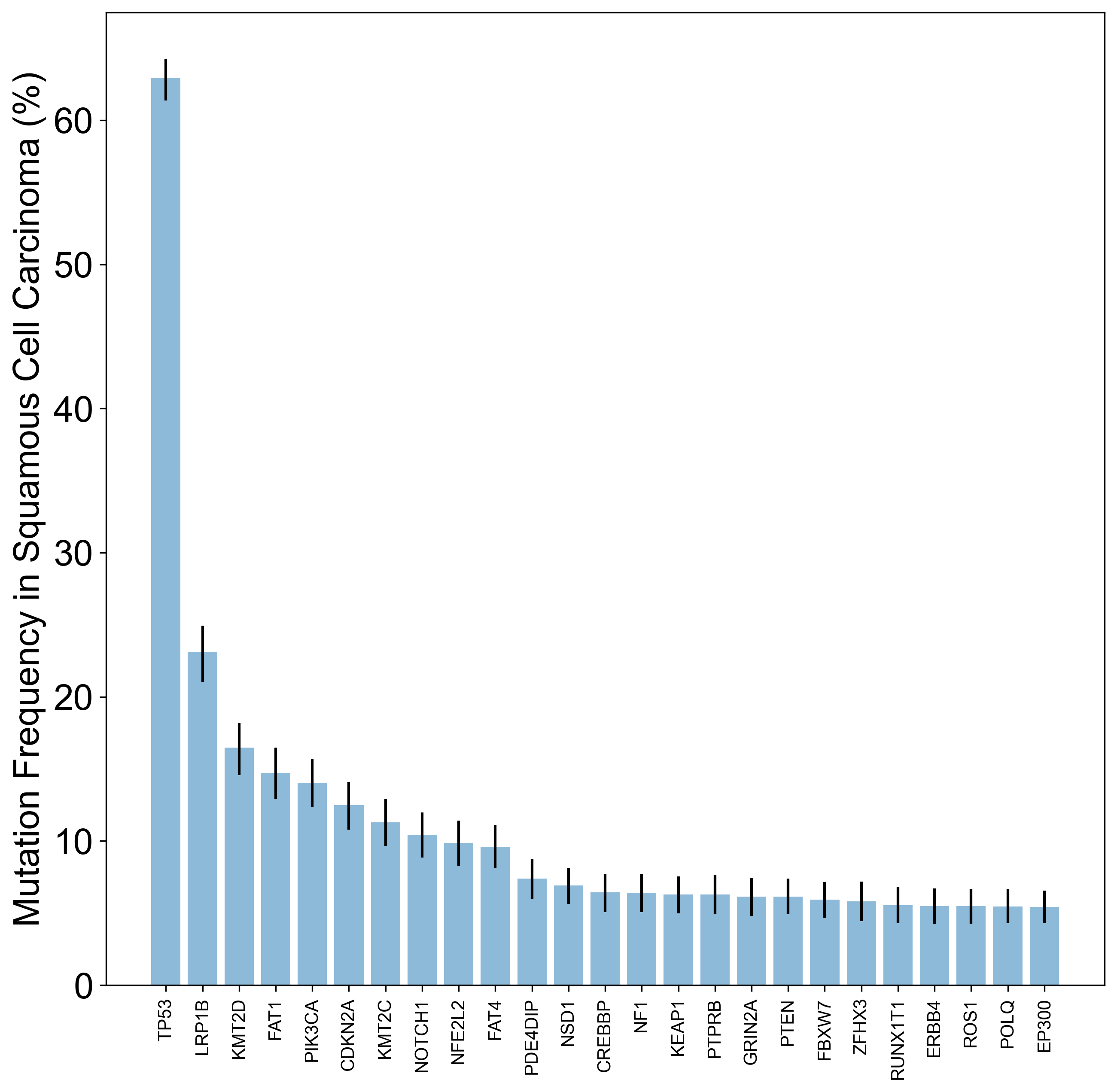

Supplement: Supplementary file 8 — Supplementary Software 1 [file 41467_2021_26213_MOESM8_ESM.zip › Supplementary Software 1/Results/Figure4E_Top25_Squamous Cell Carcinoma.png]

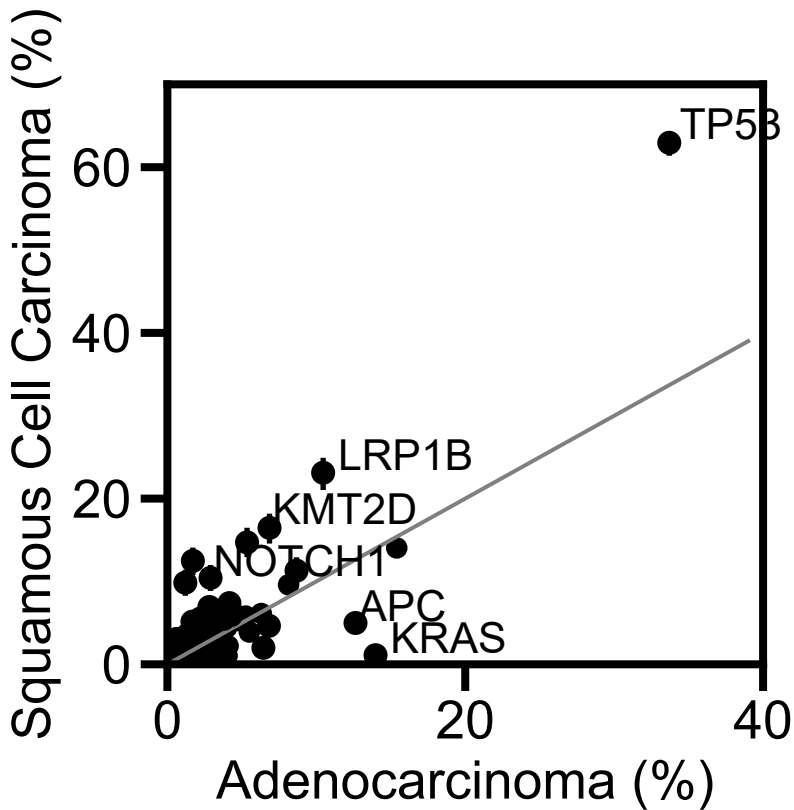

Supplement: Supplementary file 8 — Supplementary Software 1 [file 41467_2021_26213_MOESM8_ESM.zip › Supplementary Software 1/Results/Figure4F_SCCvsAdeno_scatter_CT1.pdf]

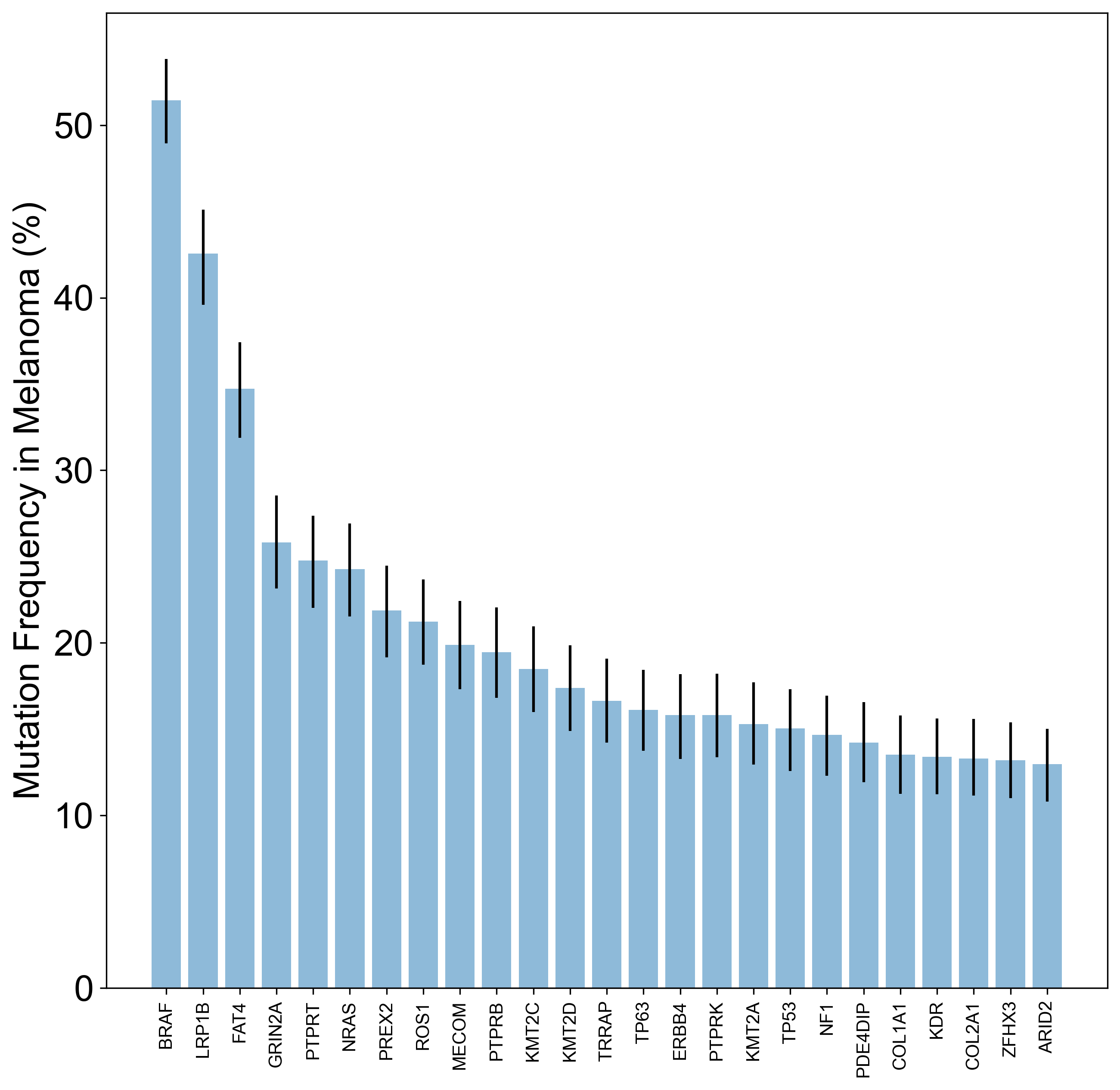

Supplement: Supplementary file 8 — Supplementary Software 1 [file 41467_2021_26213_MOESM8_ESM.zip › Supplementary Software 1/Results/Figure4G_Top25_Melanoma.png]

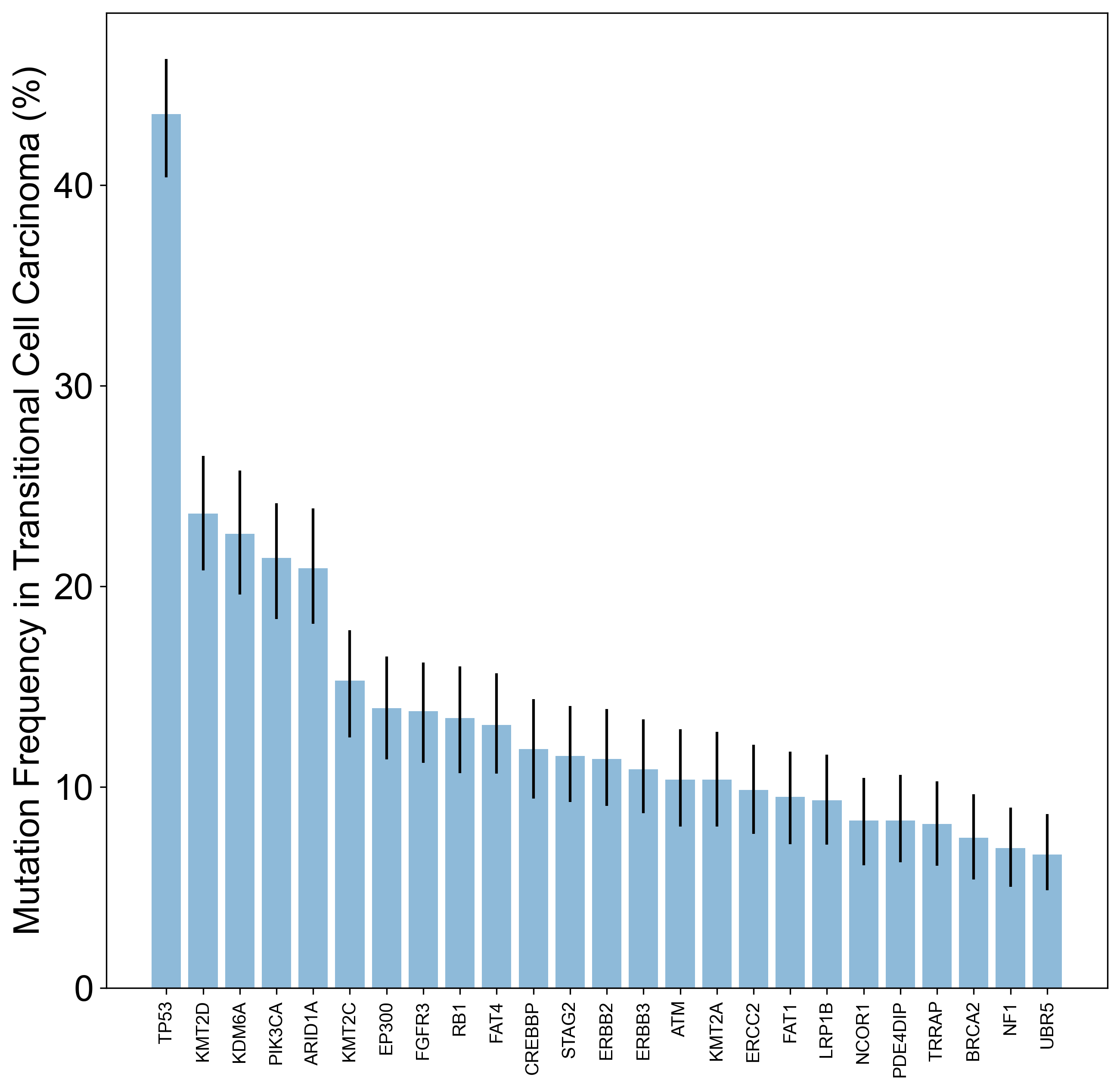

Supplement: Supplementary file 8 — Supplementary Software 1 [file 41467_2021_26213_MOESM8_ESM.zip › Supplementary Software 1/Results/Figure4H_Top25_Transitional Cell Carcinoma.png]
